# Differential regulation of humification by Maillard reaction precursors during the co-composting of pig manure and spent mushroom substrate

**DOI:** 10.1371/journal.pone.0361892

**Published:** 2026-11-11

**Authors:** Mingshuo Wang, Nan Wang, Donghui Dai, Xinrui Yang, Haibo Li, Xueqi Li, Shuai Wang

**Affiliations:** College of Agriculture, Jilin Agricultural Science and Technology College, Jilin, China; Lusofona University of Humanities and Technologies: Universidade Lusofona de Humanidades e Tecnologias, PORTUGAL

## Abstract

The rapid expansion of intensive agriculture has generated massive amounts of pig manure (PM) and spent mushroom substrate (SMS), posing severe environmental challenges. Although aerobic co-composting sustainably recycled these wastes, natural humification was typically slow, yielding immature humus (HS) with severely limited agronomic value. Therefore, this study comprehensively investigated the differential regulatory effects of distinct Maillard reaction precursors (MRPs)—glucose (Glu), catechol (Cat), and glycine (Gly)—on humification dynamics, humic acid (HA) structural evolution, and overall compost quality during a 120-day controlled mesophilic incubation experiment conducted under controlled 28 ± 0.5°C conditions, rather than traditional variable-temperature thermophilic composting systems. Chemical properties and HS fractionation were systematically analyzed alongside spectroscopic evaluations (3D-EEM, FTIR), principal component analysis (PCA), and structural equation modeling (SEM). Results demonstrated that all tested MRPs significantly mitigated initial phytotoxicity and enhanced ultimate compost maturity, achieving final T values (0.53–0.75) well below the established highly mature threshold (≤ 0.8). Specifically, Glu exhibited the strongest comprehensive regulatory effect (PCA score: 0.772, *p* < 0.05, significantly higher than Cat, Gly, and CK treatments); by stimulating microbial metabolism as inferred from DOM fluorescence properties and HS transformation dynamics, it maximized stable humin (Hu) accumulation and achieved the highest humic-extracted acid (HE) content (57.7 g/kg, significant difference relative to the other three groups at *p* < 0.05). The Cat treatment effectively alleviated salinity stress, significantly reducing electrical conductivity by a pronounced 63.5% (*p* **< 0.05, significantly lower final** EC **than** CK**, Glu, and Gly**) via phenolic oxidative coupling, while retaining the highest total intensity for aliphatic structures. Furthermore, Gly supplementation successfully facilitated the highest degree of HA aromatization, evidenced by a drastic 36.8% reduction in the H/C atomic ratio (*p* **< 0.05, the largest H/C decline among all treatments**). SEM supported our hypothesized causal framework and provided statistical evidence indicating that MRPs acted as pivotal drivers, exerting a significant direct positive effect on final compost quality by synergistically accelerating humification. These findings elucidate precursor-specific pathways of humification, providing a targeted and data-supported regulatory strategy to successfully enhance HS quality and effectively promote the high-value recycling of agricultural organic wastes.

## 1. Introduction

The rapid expansion of intensive animal husbandry and the global edible fungi industry has resulted in the excessive accumulation of pig manure (PM) and spent mushroom substrate (SMS, residual substrate remaining after mushroom fruiting‑body harvest), posing significant challenges to the global ecological environment [1]. Improper disposal of these organic wastes not only causes soil and water pollution but also wastes valuable organic resources. Agricultural organic waste co-composting represents a promising pathway for circular agriculture by converting manure and agro-residues into high-quality organic amendments, which improves resource‑use efficiency and mitigates environmental risks from improper waste disposal [2]. Aerobic co-composting is an environmentally friendly and sustainable organic waste recycling technology, which can convert PM and SMS into high-quality organic fertilizers rich in humus (HS, complex heterogeneous organic matter formed via biotic-abiotic transformation during composting) [1,3]. Humification is the transformation of simple organic substances into complex HS during composting, directly influencing their formation and properties [3]. However, the natural humification of PM-SMS co-composting is typically slow, leading to low humic acid (HA, alkali-soluble, acid-precipitable core fraction of HS with large‑molecular‑weight aromatic structures) content and immature HS structure in the final product, limiting its application potential. Thus, optimizing humification and enhancing HS quality is critical for the high-value recycling of PM and SMS, and serves as a key solution to related environmental problems, with profound implications for ecological conservation and sustainable agriculture.

The Maillard reaction, a non-enzymatic browning process between Maillard reaction precursors (MRPs), formed humic-like substances analogous to natural HS [4]. It was well recognized as a key abiotic humification pathway during composting, with extensive research on its regulatory role. Xu et al. [5] showed that ammonifying microorganism inoculation enhanced HS-related enzyme activity, reshaped the fungal community, and promoted HS formation in cow manure-wheat husk co-composting. Wang et al. [6] identified a polyphenol-Maillard synergistic pathway, where MRPs promoted polysaccharide accumulation in humic-like acid systems under high glucose (Glu), via enhanced cross-linking between MRPs and HLA precursors. Jiao et al. [7] reported that oxalic acid and phenolics enhanced HS conversion by activating lignin-degrading microorganisms. Zhu et al. [8] showed *Gloeophyllum trabeum* pretreatment in wheat straw-PM co-composting increased aromatic functional groups in HS and shortened the composting cycle. Chen et al. [9] confirmed sucrose or maltose supplementation in chicken manure-fruit/vegetable waste co-composting increased HS content by promoting MRPs-HS precursor cross-linking. Advancing such compost-driven organic‑resource recycling aligns with core goals of sustainable agricultural resource valorization [10]. Despite progress in understanding how MRPs regulate humification during organic waste co-composting, studies specifically addressing PM–SMS systems remain limited.

Few empirical studies have performed side‑by‑side comparisons of how chemically distinct MRPs alter humification progression, HA molecular structural transformation, and the overall agronomic performance within PM‑SMS co‑composting. Accordingly, it remains poorly understood whether carbon (C)‑source‑driven, phenol‑driven, and nitrogen (N)‑donor‑driven humification pathways would manifest divergently for this common agricultural waste combination. Given their distinct chemical structures and functional groups, we put forward the following working hypothesis for this study: that Glu, catechol (Cat), and glycine (Gly), as representative sugar-type, phenolic-type, and amino acid-type Maillard reaction precursors, are anticipated to trigger partially divergent humification trajectories in PM‑SMS co-composting. More specifically, (i) Glu, as an easily fermentable reducing‑sugar C source, would stimulate microbial metabolic activity and supply C skeletons for Maillard-type reactions, tending to yield better overall compost performance via enhanced accumulation of stable HS fractions; (ii) Cat, as a typical phenolic precursor, would mainly drive abiotic phenolic oxidative‑coupling reactions, mitigate salinity-related phytotoxicity through salt complexation, and preserve aliphatic structural moieties of HA rather than strongly promoting aromatic condensation; (iii) Gly, an amino‑group-containing N-rich precursor, would provide N substrates for Maillard condensation and favour the generation of N-containing aromatic heterocycles, thereby improving the aromatization degree of HA. Therefore, a 120-day controlled mesophilic incubation was conducted using Glu, Cat, and Gly to compare their effects on humification in PM–SMS co-compost. A comprehensive analytical strategy, including chemical properties detection, HS composition, three-dimensional excitation–emission matrix (3D-EEM) fluorescence spectroscopy, Fourier-transform infrared (FTIR) spectroscopy, and principal component analysis (PCA), was employed to characterize humification succession and HA structural variation. This study aims to optimize the humification process via the addition of different MRPs, assess the impacts of MRPs on humification product quality and HA structural dynamics, and reveal the intrinsic mechanisms of MRPs in facilitating humification during PM–SMS co-composting.

## 2. Materials and methods

### 2.1. Raw materials

Two primary raw materials, SMS and PM, were collected from the on-campus experimental farm of Jilin Agricultural Science and Technology College (Jilin, China). SMS was the residual cultivation substrate after *Pleurotus ostreatus* harvest, with the original formulation (dry weight basis): 60% hardwood sawdust, 28% corn cob, 10% wheat bran, and 2% lime. Chemical properties of air-dried SMS and PM were as follows: SMS: total organic C (TOC) 50.0%, total N (TN) 0.4%, total phosphorus (TP) 0.90%, total potassium (TK) 0.28%, pH 5.45; PM: TOC 2.53%, TN 4.85%, TP 1.81%, TK 3.26%, pH 7.65. Before the experiment, SMS was air-dried and cut into 0.5–1.0 cm pieces to maintain porosity, while PM was ground and sieved (2 mm) to ensure uniform particle size. Calcium bentonite (≥98% purity) was purchased from Sinopharm Chemical Reagent Co., Ltd. (Shanghai, China). A commercially available liquid microbial inoculum (Tonghua Wanying Biotechnology Co., Ltd., Jilin Province, China) comprised the following six functionally characterized strains: *Trichoderma harzianum*, *Bacillus subtilis*, *Bacillus licheniformis*, *Aspergillus niger*, *Aspergillus oryzae*, and *Saccharomyces sp.* The product exhibited a viable cell concentration of ≥ 2 × 10^6^ CFU/mL and a pH range of 3.0–4.5. Immediately prior to application, the inoculum was aseptically diluted 100-fold with sterile deionized water to yield a working suspension containing 2 × 10^4^ CFU/mL.

### 2.2. Experimental design

The 120-day aerobic incubation experiment was designed to simulate the mesophilic phase of conventional PM‑SMS co‑composting, and was conducted under strictly controlled constant temperature conditions (28 ± 0.5°C; 40 ± 2% relative humidity). Unlike conventional aerobic composting that undergoes a complete thermal succession including a high-temperature thermophilic stage, a constant mesophilic temperature of 28°C was deliberately employed in this study to eliminate temperature-related confounding variables. This targeted experimental design enabled us to specifically disentangle humification mechanisms governed by mesophilic microorganisms and MRP-driven abiotic pathways, without the interference of drastic thermal fluctuations and thermophilic microbial succession typical of practical full-scale composting.. Samples were collected at 0, 30, 60, 90, and 120 days. The incubation mixture for simulating co‑composting was prepared by mixing SMS and PM at a 1:1 (w/w) ratio, with each batch adjusted to 1.0 kg. 4.0 g of calcium bentonite (0.4% of total mixture weight) was added to each treatment to enhance structural stability, water retention, and HS precursor polymerization. The experiment included three treatment groups and a control (CK), with three replicates per group for reproducibility. Grouping details: Cat group (100 mL of 0.28 mol/L catechol solution added to SMS–PM mixture), Glu group (100 mL of 0.28 mol/L glucose solution), Gly group (100 mL of 0.28 mol/L glycine solution), and CK group (100 mL of deionized water instead of organic substrates). This equal-molar concentration (0.28 mol/L) was selected to allow fair comparison of humification pathways triggered by three chemically different MRPs, instead of screening for practically optimal application dosage. This molar level has been widely adopted in abiotic humification simulation studies for MRPs [4]. Additionally, 50 mL of 0.25 mol/L (NH_4_)_2_SO_4_ solution and deionized water were added to adjust the initial moisture content to 65%. The mixtures were transferred into high-density polyethylene containers (diameter: 30 cm; height: 15 cm) as independent reactors. Each reactor was inoculated with 25 mL of diluted microbial inoculum (5 × 10^5^ CFU/reactor) and sealed with a gas-permeable membrane (0.22 μm pore size; Millipore, Billerica, MA, USA) for gas exchange and contamination prevention. All reactors were incubated in a temperature-controlled chamber (Model SPX-250B, Shanghai Boxun Industrial Co., Ltd., China) at 28°C under aerobic conditions, with manual turning every two weeks using a sterile spatula. At each sampling point, ≥ 30 g of material was collected from each reactor via five-point sampling (center and four corners at 5 and 10 cm depths). Sub-samples from each reactor were homogenized into a composite sample, split into two portions: one stored at 4°C for immediate dissolved organic matter (DOM) and electrical conductivity (EC) analysis, and the other air-dried for subsequent nutrient and HS composition determination. Deionized water was weekly replenished based on weight loss to maintain 65% moisture for consistent conditions throughout the entire incubation period.

### 2.3. Determination of chemical properties

Incubation samples were air-dried at 60°C for 48 h, ground, and sieved through a 0.15 mm nylon sieve. The contents of TN, TP, and TK were determined using standardized analytical methods: TN was quantified via the Kjeldahl method with a Kjeldahl analyzer (Model K9840, Hanon Instruments, Jinan, China), which involved digestion in concentrated H_2_SO_4_ catalyzed by a CuSO_4_–K_2_SO_4_ mixture. TP was measured by ultraviolet spectrophotometry (Model UV-2600, Shimadzu, Kyoto, Japan) at a wavelength of 700 nm following sample digestion with a concentrated H_2_SO_4_–H_2_O_2_ mixture (v/v = 5:1). TK was analyzed using flame photometry (Model FP640, Shanghai INESA Analytical Instrument Co., Ltd., Shanghai, China) after digestion with the same H_2_SO_4_–H_2_O_2_ mixture as used for TP determination. organic matter (OM) and TOC contents were determined via the K_2_Cr_2_O_7_ volumetric method under external heating (170–180°C). TOC was calculated as OM × 0.58, where 0.58 was the established conversion factor for OM in agricultural waste. For aqueous extraction, 10 g of fresh compost sample was mixed with 100 mL of deionized water at a solid-to-liquid ratio of 1:10 (w/v) and shaken on a horizontal reciprocating shaker (Model SHZ-88, Taizhou Honghua Instrument Co., Ltd., Taizhou, China) at 25 ± 1°C and 180 ± 5 rpm for 1 h. The resulting suspension was centrifuged at 2264 × g for 10 min (Model XJ-202, Shanghai Anting Scientific Instrument Factory, Shanghai, China), and the supernatant was filtered through a 0.45 μm hydrophilic polyethersulfone membrane (Millipore, Billerica, MA, USA) to obtain compost leachate. EC and germination index (GI) were quantified to evaluate phytotoxicity and maturity degree of the humification products, respectively. EC was determined at 25°C using a DDS-307A conductivity meter (Shanghai Precision & Scientific Instrument Co., Ltd., Shanghai, China), with three replicate measurements performed for each sample. For GI assessment, 10 mL of compost leachate was pipetted into sterile 9 cm Petri dishes, each lined with two layers of Whatman No. 1 qualitative filter paper (GE Healthcare, Chicago, IL, USA). Ten healthy, disease-free cucumber seeds (*Cucumis sativus* L.) of uniform size were evenly placed in each dish using sterile forceps. Deionized water was used as the CK, which was treated identically to all experimental groups. All treatments, including the CK, were conducted in triplicate. The Petri dishes were then incubated in the dark at 25 ± 1°C for 48 h. GI was calculated using the following equation:


$$\begin{array}{c} \text{GI}=\frac{\text{R }\times \text{ L}}{{\text{R}}_{\text{0}}\;\times \;{\text{L}}_{\text{0}}}\times 100\% \end{array}$$

where R and L are germination rate (%) and average root length (mm) of seeds treated with compost leachate, respectively; R_0_ and L_0_ are germination rate (%) and average root length (mm) of seeds in deionized-water control group. The T was an indicator used to evaluate the maturity of compost. Its core definition was: T value = (final C/N ratio of compost)/(initial C/N ratio of compost). *A T‑value* ≤ *0.8 indicates sufficient* OM *decomposition* [11].

### 2.4. Extraction and C content determination of HS fractions

Humic acid (HA), fulvic acid (FA), humic-extracted acid (HE), and humin (Hu) were extracted from the incubation samples according to the method reported by Zhou et al. [12]. The C contents of water-soluble substances (WSS), HE, HA, and Hu were determined and denoted as C_WSS_, C_HE_, C_HA_, and C_Hu_, respectively. The C content of FA (C_FA_) was subsequently calculated as the difference between C_HE_ and C_HA_ (i.e., C_FA_ = C_HE_ − C_HA_). The degree of polymerization (DP), a key indicator reflecting the compost humification degree, was calculated using the formula: DP = C_HA_ / C_FA_ [13]. For further structural characterization, the HA solution was purified by electrodialysis (Model EDR-2, Lvjing Environmental Protection Technology Co., Ltd., Shanghai, China) to eliminate residual inorganic salts, and then freeze-dried (Model FD-1A-50, Boyikang Laboratory Instrument Co., Ltd., Beijing, China) to yield solid HA powder. The resulting HA powder was homogenized with a planetary ball mill (Model XQM-4, Instrument Factory of Nanjing University, Nanjing, China) and subsequently sieved through a 0.01 mm nylon mesh sieve. Elemental compositions (C, H, N, and O) of the solid HA powder were analyzed using an elemental analyzer (Model PE 2400 II CHNS/O, PerkinElmer Inc., Waltham, MA, USA), with atropine (C_17_H_23_NO_3_) serving as the calibration standard. Fourier transform infrared (FTIR) spectra were acquired on an FTIR spectrometer (Model FTIR-850, Gangdong Science and Technology Development Co., Ltd., Tianjin, China) in the wavenumber range of 400–4000 cm^-1^ at a spectral resolution of 4 cm^-1^, with 32 scans per sample to improve the signal-to-noise ratio. The functional groups of the HA samples were identified using the dedicated FTIR 850 software.

### 2.5. Three-dimensional excitation-emission matrix (3D-EEM) of DOM

Fresh incubation samples (5.0 g) were homogenized with distilled water at a solid-to-liquid ratio of 1:30 (w/v), then incubated in a temperature-controlled shaker at 25 ± 1°C with continuous shaking for 24 h. The mixture was centrifuged at 12,879 × g for 15 min using the high-speed centrifuge described in the HS extraction section, and the supernatant was vacuum-filtered through a 0.45-μm hydrophilic polyethersulfone membrane (Millipore, Billerica, MA, USA). The filtrate was stored at 4°C and analyzed within 24 h to preserve DOM stability. 3D-EEM fluorescence spectra were acquired on a F-7000 fluorescence spectrophotometer (Hitachi High-Technologies Corporation, Tokyo, Japan), with the optimized parameters: E_x_ wavelength range 200–550 nm (step interval: 5 nm), E_m_ wavelength range 250–550 nm (step interval: 2 nm), E_x_/E_m_ slit width 5 nm, and scanning speed 2400 nm/min. Rayleigh and Raman scattering interferences were eliminated by subtracting the 3D-EEM spectrum of ultrapure water from each sample spectrum, and the processed spectra were visualized using MATLAB 2020 (MathWorks, Natick, MA, USA). Three characteristic fluorescence indices were calculated for DOM characterization: fluorescence index (FI) (E_x_ 370 nm, ratio of E_m_ 470 nm to 520 nm fluorescence intensity), autochthonous contribution (BIX) (E_x_ 310 nm, ratio of E_m_ 380 nm to 430 nm fluorescence intensity), and humification index (HIX) (E_x_ 260 nm, ratio of integrated fluorescence intensity at E_m_ 435–480 nm to that at Em 330–375 nm) [14].

### 2.6. Statistical analyses

All experimental data **were processed** and **visualized** using Origin 2021 software (OriginLab Corporation, Hampton, MA, USA). Linear mixed‑effects models (LMM) were applied to evaluate main effects of MRP amendment, incubation time, and their interaction for humification‑related longitudinal parameters. MRP treatment and incubation time were defined as fixed‑effect factors, and individual reactor ID was included as a random intercept to account for temporal autocorrelation caused by repeated destructive subsampling from identical incubation reactors across multiple sampling days. One-way analysis of variance (ANOVA) **was conducted** with SPSS 20.0 software (IBM Corporation, Armonk, NY, USA) to evaluate significant differences among different treatment groups at individual fixed sampling time points, followed by post-hoc multiple-comparison analysis. Statistical significance was **set** at a probability level of *p* < 0.05. Data were **presented** as mean ± standard deviation (SD) based on three independent replicates. In the bar charts, SD values **were displayed** as error bars to indicate the degree of variability in the experimental data. PCA was conducted to evaluate the contribution of each variable to the measured parameters. Prior to PCA, all input variables were standardized via Z-score transformation to eliminate dimensional differences among indicators. Variable selection included total nutrient content, OM, CWSS, CHE, CHA, CHu, EC, GI, DP, C/N ratio, O/C ratio, H/C ratio, and [(a + b + c)/d] (a total of 13 variables). PCA and PLS-SEM were performed on observations collected at the final incubation time point (day 120). A total of 12 independent observations (4 treatments × 3 biological replicates) were entered into both PCA and PLS‑SEM, rather than treatment‑level averaged values. The Kaiser-Meyer-Olkin (KMO) test and Bartlett’s sphericity test were implemented to verify data suitability for PCA; the calculated KMO statistic was 0.677, and Bartlett’s sphericity test yielded χ2 = 580.998, df = 78, *p* < 0.001, confirming the dataset satisfied the basic prerequisites for principal component extraction. PCA was performed using SPSS AU (Beijing Qingxi Technology Co., Ltd, Beijing, China). The first three principal components cumulatively explained 99.999% of the total variance, approaching 100%, which arises because 13 input variables were computed from 12 independent samples, leading to complete description of sample variance within the extracted three components. For PLS-SEM, significance testing of standardized path coefficients was implemented via bootstrap resampling (n = 1000 bootstraps). We emphasize that bootstrap resampling cannot overcome constraints of the relatively small sample size (N = 12); therefore, all path coefficients should be interpreted as exploratory correlational findings rather than definitive causal proof. Considering the limited biological sample size, path coefficients are exploratory outcomes, and their stability is conditional on the current dataset. The structural equation model and its corresponding standardized path coefficient diagram were generated using the R package plspm.

## 3. Results and discussion

### 3.1. Effects of MRPs addition on fluorescence indices of DOM during simulated mesophilic co-composting incubation

DOM represents a highly active fraction during OM humification, providing readily degradable substrates that fuel microbial metabolism and accelerate humification maturation [15].

As depicted in Fig 1 and S1 Table, FI values ≤ 1.4 indicate a terrestrial plant-derived DOM origin, values ≥ 1.9 indicate a microbial origin, and intermediate values (1.4–1.9) reflected a mixed contribution [16]. At the initial stage, the FI values for all treatments were within the 1.4–1.9 range, indicating an initially mixed DOM source. By day 120, the FI values in the CK and Cat treatments had increased to 1.91 and 2.03, respectively. By exceeding the 1.9 threshold, their DOM sources had fundamentally shifted from a mixed origin to a predominantly microbial origin. This observation was particularly evident in the Cat treatment, where the ortho-dihydroxyl moiety of catechol raised the hypothetical possibility that it favored the proliferation of specific phenol-degrading bacteria, which might potentially contribute to microbial DOM generation and push the FI value beyond the microbial threshold [17,18]. In contrast, the Glu and Gly treatments maintained their mixed-source characteristics throughout the entire 120-day mesophilic incubation process. Although Glu was hypothesized to have functioned as a highly labile C source that stimulated microbial metabolism and increased the FI from 1.46 to 1.79, the final value had strictly remained within the intermediate range [19]. Similarly, Gly was the only treatment showing a FI decrease (from 1.51 to 1.42). Although glycine could supply both C and N, its C fraction existed in amino‑bound form with relatively low bioavailability compared with readily fermentable sugars. It was hypothesized that this limited bioavailability of Gly-derived C, rather than a total absence of C, constrained overall heterotrophic microbial activity and triggered partial DOM mineralization [20,21]; however, its final FI remained within the intermediate 1.4–1.9 range. Therefore, while Glu and Gly significantly regulated microbial metabolic intensity (causing internal FI fluctuations), they did not alter the fundamental mixed-source nature of the DOM. The overall temporal trends of BIX were broadly consistent with those of FI across treatments, both reflecting treatment-specific shifts in DOM sources and microbial activity; however, discrepancies existed in relative increase magnitudes due to differences in initial baseline values. By day 120, the BIX value of the Cat treatment surged by 96.2% relative to its initial value, reaching 0.469. Concurrently, the CK and Glu treatments experienced BIX increases of 103.5% and 94.4%, respectively, whereas the Gly treatment recorded a marginal decrease of 1.2%. Notably, although CK showed the highest relative BIX increase, its initial BIX baseline (0.171) was markedly lower than that of the Cat treatment (0.239); accordingly, the final absolute BIX value in the Cat treatment remained significantly higher than that in the CK (*p* < 0.05), which supported the premise that Cat amendment enhanced microbial DOM production, while the elevated final BIX value in the Cat group was hypothesized to arise from the synergistic effects of metabolic substrate provision and consequent potential shifts in microbial community composition [17].

**Fig 1:**
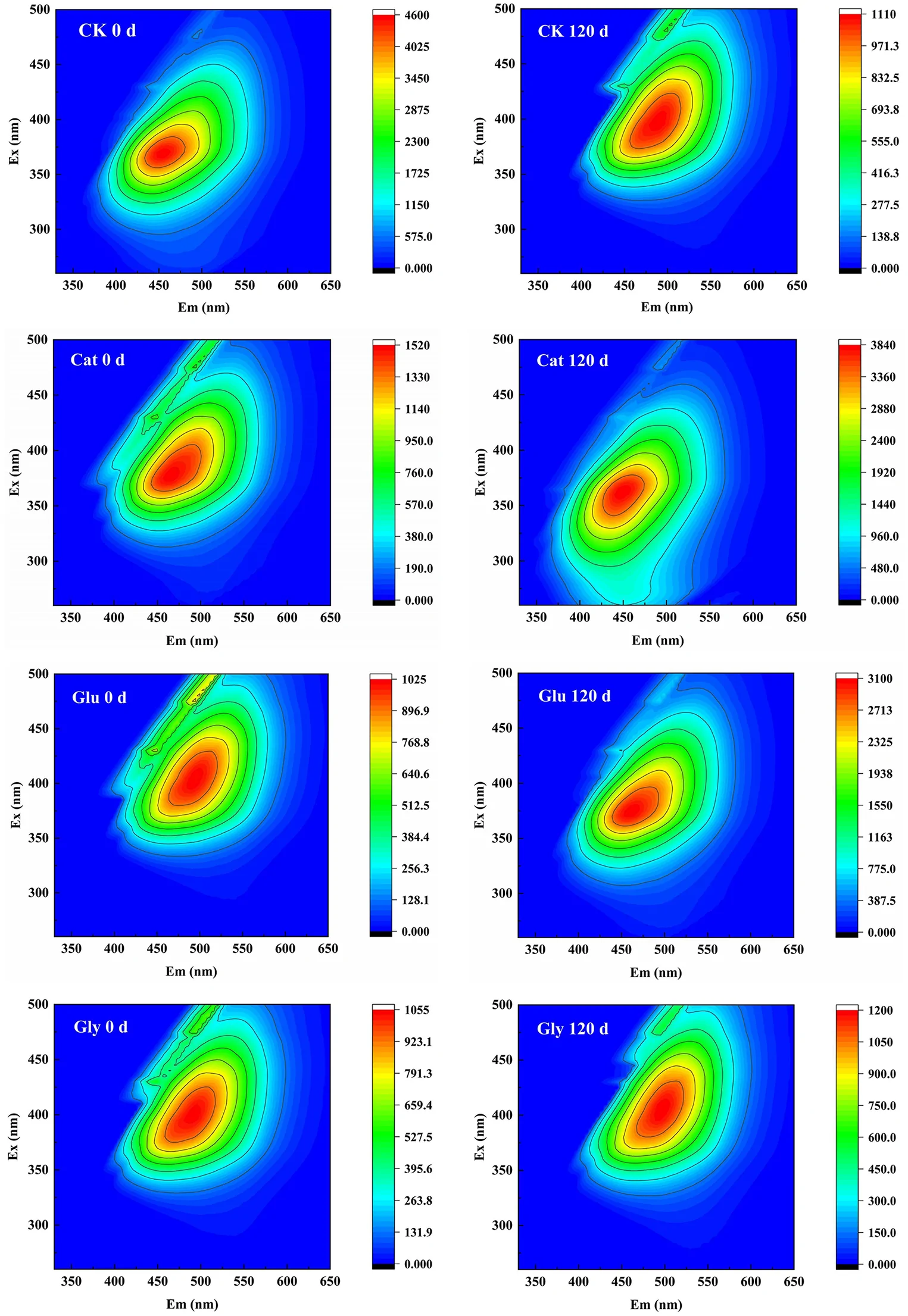
3D-EEM fluorescence spectra of DOM obtained at the initial and final stages of simulated mesophilic co-composting incubation. Note: Contour colors denote fluorescence intensity (arbitrary units, a.u.): warmer colors (red/yellow) indicate higher intensity, and cooler colors (blue) indicate lower intensity.

In contrast, the BIX increase in the Glu treatment **was proposed to be** primarily fueled by enhanced microbial biomass accumulation triggered directly by exogenous C supplementation [17]. The negligible BIX decline in the Gly treatment suggested that the prevailing C limitation established a dynamic equilibrium between the synthesis and consumption of autochthonous DOM. Over the 120-day period, the HIX increased by 72.2% in the CK, 112.4% in the Glu treatment, and 46.5% in the Gly treatment. Interestingly, the HIX of the Cat treatment exhibited a decline from 9.2 down to 5.8. Serving as a core MRP, Glu was hypothesized to optimize the microbial microenvironment by providing abundant labile C and essential C skeletons; this directed might potentially modulate microbial metabolism toward continuous humification, ultimately driving the highest accumulation of stable aromatic compounds [17,22]. Notably, this inference was drawn from indirect chemical and spectroscopic evidence rather than direct microbial measurements, and thus remained mechanistically speculative. The moderate HIX increase observed in the Gly treatment was likely due to its -NH_2_ groups providing supplementary N, which marginally enhanced microbial decomposition efficiency [23]. Conversely, the apparent HIX decline in the Cat treatment was hypothesized to fundamentally stem from phenol-degrading bacteria preferentially catabolizing phenolic substrates, which was proposed to sequester microbial metabolic resources and limit secondary resource allocation to newly synthesized DOM humification [17].

### 3.2. Effects of MRPs addition on EC during simulated mesophilic co‑composting incubation

EC is a key indicator of humification product safety and agronomic suitability [24]. At the onset of the incubation experiment (day 0), the EC values across the four treatments ranged from 2.65 to 3.15 mS/cm (Fig 2). Following the 120-day mesophilic incubation period, EC exhibited treatment-specific trajectories: the CK showed a significant increase, which was consistent with the typical accumulation of soluble salts during unamended natural mesophilic OM decomposition. Conversely, the Cat treatment experienced a pronounced 63.5% reduction in EC, yielding the lowest final value; the Glu treatment displayed a moderate 23.0% increase; and the Gly treatment remained relatively stable with only marginal EC fluctuations. Notably, all MRP treatments maintained lower EC levels than the CK and remained below the 4.0 mS/cm phytotoxicity threshold throughout the process. This indicated that MRPs effectively mitigated soluble salt accumulation and enhanced the agronomic safety of the final humification products [24]. These divergent EC dynamics could be attributed to the distinct biochemical mechanisms underlying each specific MRP [6].

**Fig 2:**
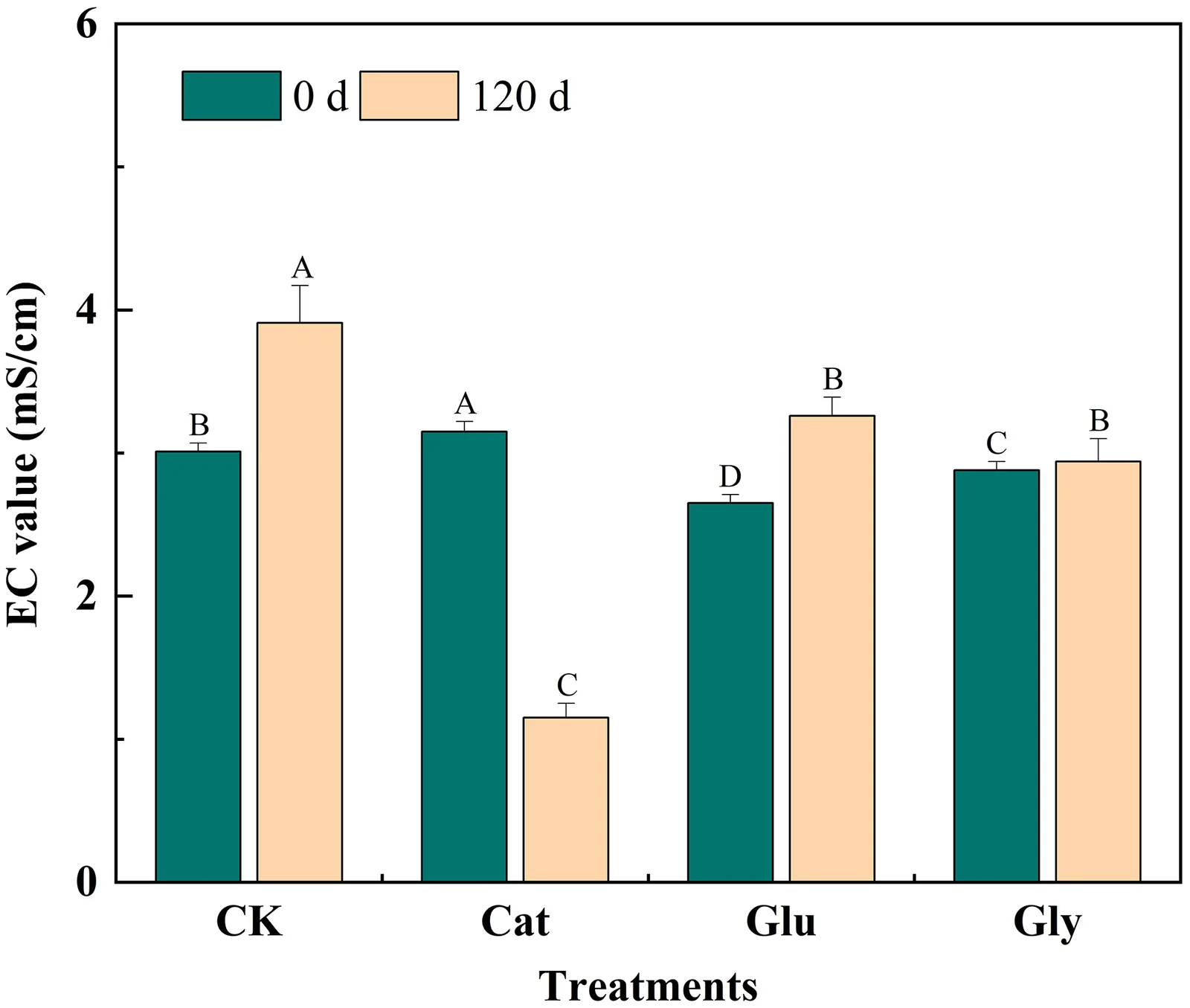
Variations in EC during simulated mesophilic PM-SMS co-composting incubation. Note: Error bars represent standard deviation (n = 3). Uppercase letters denote statistically significant differences among treatments at the same composting stage (*p* < 0.05).

### 3.3. Effects of MRPs addition on GI during simulated mesophilic co‑composting incubation

With respect to GI, all treatments followed an identical temporal pattern: an initial decline, a peak at day 60, and a subsequent gradual decrease (Fig 3). The initial GI decline resulted from the accumulation of phytotoxic intermediates during rapid microbial growth; the subsequent peak in GI fundamentally resulted from the degradation of these toxic substances by a stabilized microbial community; finally, the later decline was likely driven by the exhaustion of labile OM, the subsequently weakened microbial detoxification capacity, and the potential accumulation of soluble salts or excessive nutrient mineralization during the late stage [25]. Notably, on day 0, the GI of all MRP-treated incubation systems was higher than that of the CK, indicating that MRPs conferred an immediate improvement to the microbial microenvironment and mitigated initial phytotoxicity. The Glu treatment exhibited the highest peak GI, which aligned with Glu’s role as a readily assimilable C and energy source that was hypothesized to actively enhance microbial metabolic activity and thereby accelerate detoxification [17]. This interpretation was based on indirect physiological indicators and requires further validation via direct microbial assays. The Cat treatment displayed a moderate GI peak, a phenomenon hypothesized to be linked to the potential enrichment of phenol-degrading bacteria that was proposed to effectively mineralize volatile phytotoxic compounds [17]. In contrast, although the Gly treatment yielded a lower peak GI, it achieved the highest final GI among all MRP treatments. This distinct pattern suggested that mild C limitation sustained microbial metabolic stability and successfully suppressed the secondary re-formation of phytotoxic substances during the maturation phase. Ultimately, on day 120, the GI of the MRP-treated incubation products remained elevated relative to the CK, confirming the persistent ameliorative effect of MRPs on phytotoxicity during mesophilic OM humification.

**Fig 3:**
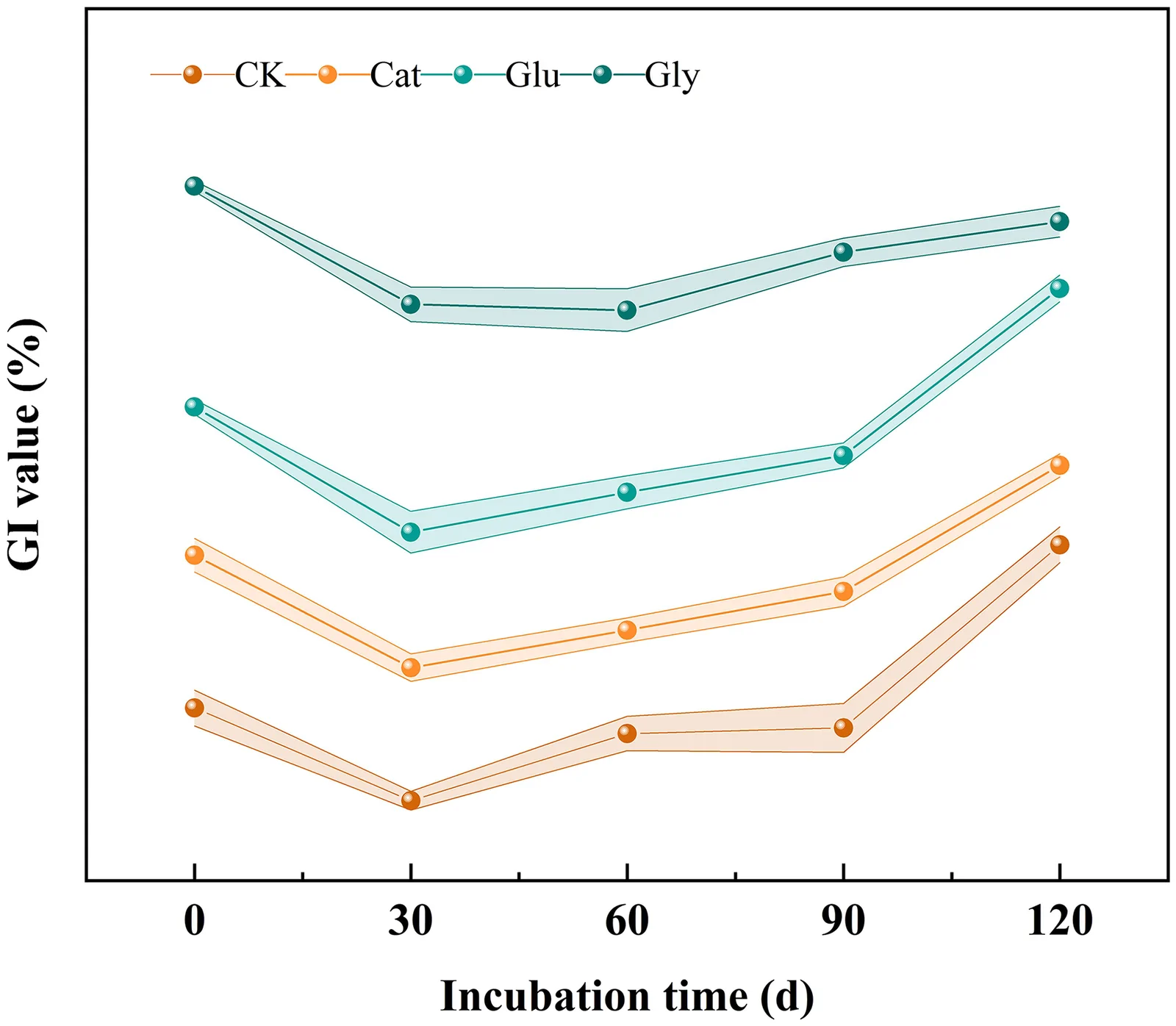
Variations in GI during simulated mesophilic PM-SMS co-composting incubation. Note: Error bars represent standard deviation (n = 3). Uppercase letters denote statistically significant differences among treatments at the same composting stage (*p* < 0.05).

### 3.4. Effects of MRPs addition on TN, TP, TK, and total nutrients during simulated mesophilic co‑composting incubation

The application of MRPs significantly altered the nutrient composition and transformation dynamics during both the initial and maturation stages of the simulated mesophilic incubation. At day 0, the contents of TN, TP, TK, and total nutrients in the MRP treatments were generally higher than those in the CK (Fig 4). This initial elevation was likely due to the immediate physicochemical integration of the organic MRP solutions into the incubation matrix, which optimized the initial substrate conditions. After 120 days, nutrient dynamics diverged markedly across treatments: the relative TN content increased in the CK, Cat, and Gly treatments, whereas the Glu treatment experienced a 13.4% decrease. The enhanced TN retention in the Gly treatment can be attributed to its -NH_2_ groups, which effectively stabilize ammonium ions [20]. Conversely, the TN decline in the Glu treatment likely resulted from an intense initial burst of microbial activity fueled by the highly labile C source; this hyperactive early phase accelerated initial ammonia volatilization prior to substantial microbial N immobilization [17]. TP was universally enriched across all treatments at maturity. This enrichment is primarily driven by the concentration effect resulting from extensive OM degradation and mass loss, alongside the role of calcium bentonite in forming stable phosphate precipitates and the potential stimulation of putative phosphorus-mineralizing bacteria by MRPs. Interestingly, the measured TK exhibited slight reductions across treatments, which was likely due to K^+^ intercalation and strong specific adsorption by the bentonite lattice, partially rendering the K unextractable by conventional digestion methods [26]. Driven by the dominant TP enrichment and partial TN increases, the total nutrient content robustly increased in all treatments at maturity.

**Fig 4:**
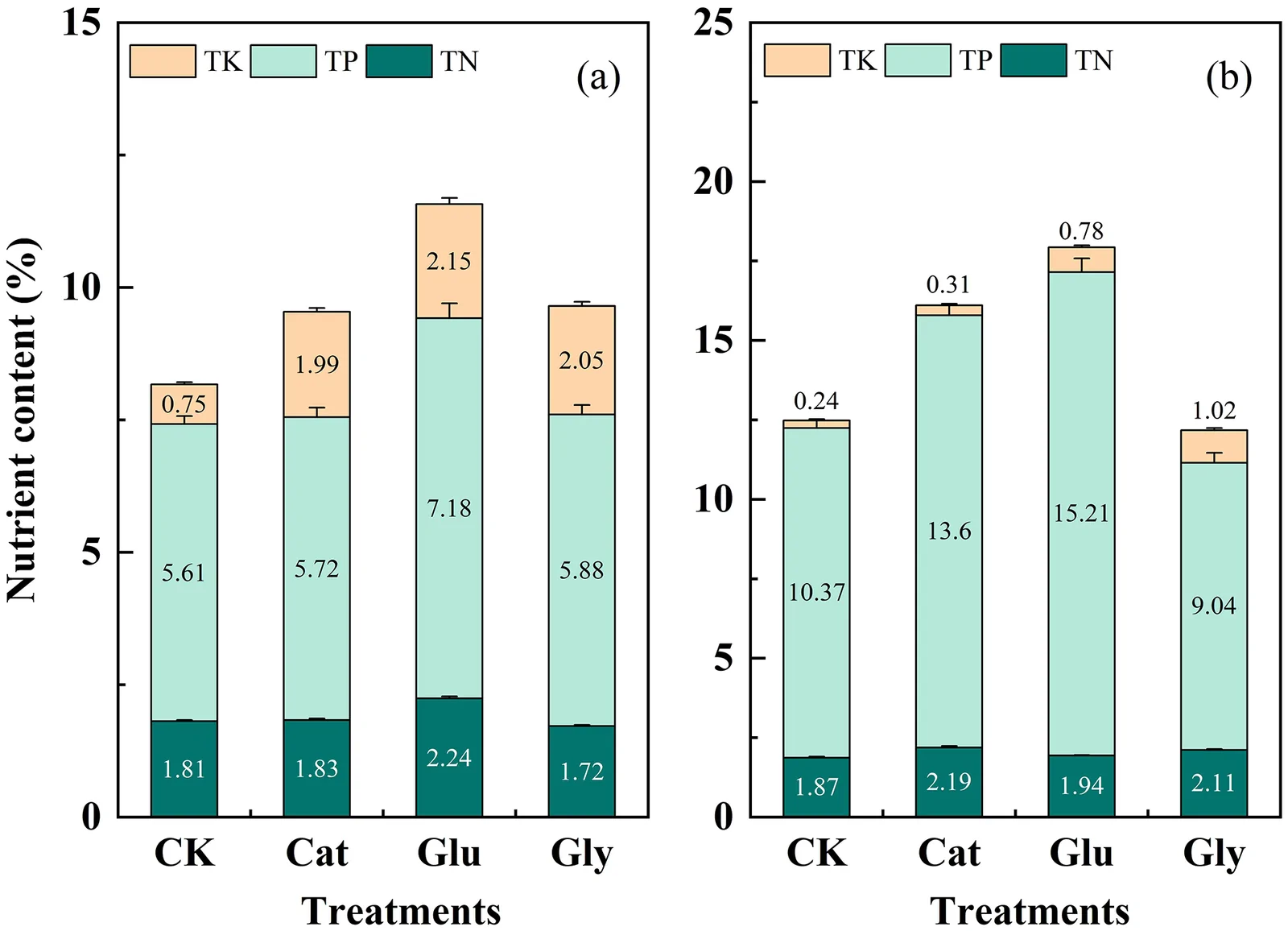
Variations in TN, TP, TK, and total nutrient contents during simulated mesophilic PM-SMS co-composting incubation. Note: (a) TN, TP, and TK contents at the initial stage of co-composting; (b) TN, TP, and TK contents at the final stage of co-composting. Error bars represent standard deviation (n = 3). Uppercase letters denote statistically significant differences among treatments at the same composting stage (*p* < 0.05).

### 3.5. Effects of MRPs addition on OM during simulated mesophilic co‑composting incubation

The degradation of OM and the progressive reduction of the C/N ratio are fundamental hallmarks of OM maturation during humification, reflecting robust microbial OM mineralization and balanced C-N utilization. All treatments exhibited a substantial decrease in OM content by the maturation stage (Fig 5). The Glu treatment demonstrated the most pronounced OM reduction (35.3%), as the readily available Glu was proposed to potently stimulate microbial metabolic activity, thereby accelerating the mineralization of indigenous OM via priming effects [27]. Conversely, the Cat treatment exhibited the mildest OM decomposition, a phenomenon attributable to its phenolic –OH groups, which actively trigger the cross-linking of organic fractions into non-degradable polymers, effectively preserving bulk OM [17].

**Fig 5:**
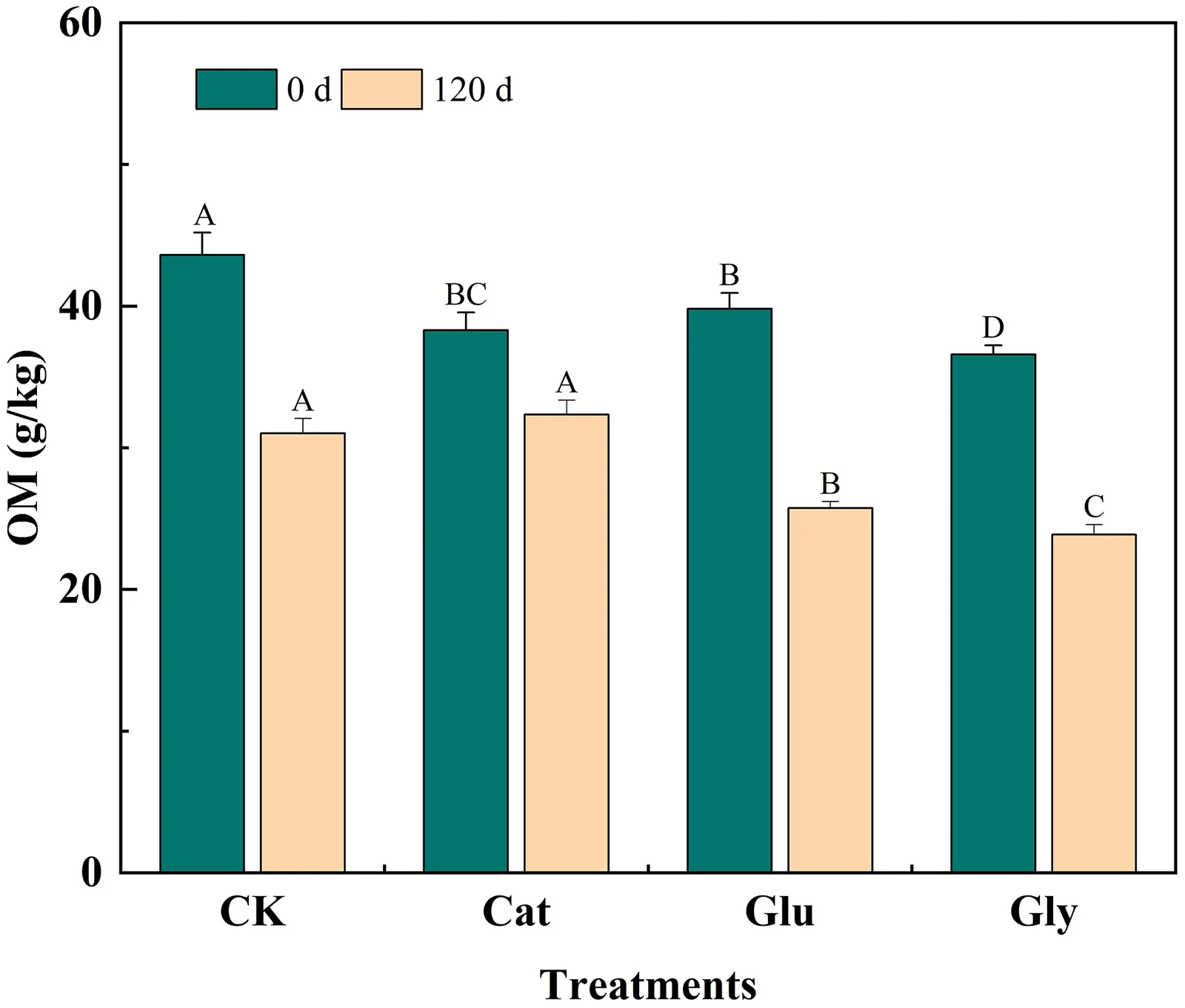
Variations in OM contents during simulated mesophilic PM-SMS co-composting incubation. Note: Error bars represent standard deviation (n = 3). Uppercase letters denote statistically significant differences among treatments at the same composting stage (p < 0.05).

### 3.6. Effects of MRPs addition on C/N ratio and T value during simulated mesophilic co‑composting incubation

Consequently, the C/N ratio decreased in all treatments by day 120 (Fig 6). The Gly treatment exhibited a substantial reduction in the C/N ratio, primarily driven by early C limitation that forced the intensive degradation of indigenous C. In contrast, the mild C/N reduction in the Glu treatment resulted from its inherently low initial C/N ratio and an optimized dynamic balance between C mineralization and N immobilization under a sufficient exogenous C supply [17]. A T value of ≤ 0.8 is widely recognized as the critical threshold for highly mature OM after composting, indicating comprehensive microbial C mineralization and N stabilization [9,28]. The final T values ranked as follows: Gly (0.53) <CK (0.69) <Cat (0.71) <Glu (0.75). Crucially, all treatments met this highly mature standard within the 120-day mesophilic incubation period, demonstrating that while MRPs differentially regulated the trajectory and degree of maturation, they fundamentally facilitated the overall maturation process.

**Fig 6:**
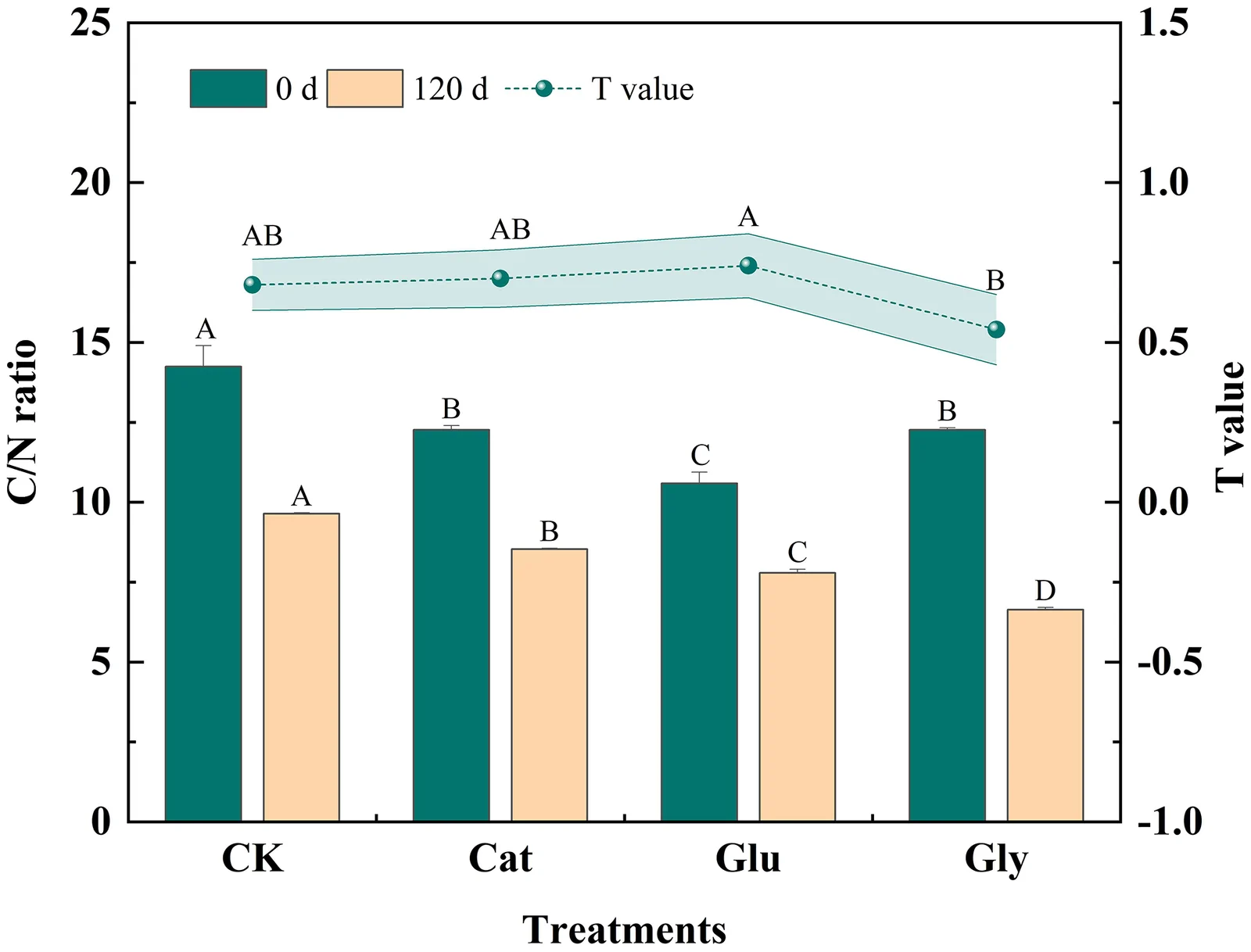
Variations in C/N ratios and T values during simulated mesophilic PM-SMS co-composting incubation. Note: Error bars represent standard deviation (n = 3). Uppercase letters denote statistically significant differences among treatments at the same composting stage (*p* < 0.05).

### 3.7. Effects of MRPs addition on C_WSS_ during simulated mesophilic co‑composting incubation

WSS represents a highly labile organic fraction and serves as the primary C source fueling initial microbial metabolism [14]. Throughout the 120-day mesophilic incubation period, C_WSS_ across all treatments exhibited a continuous decline, which directly reflected the sustained microbial degradation and utilization of labile organic C (Fig 7). At day 0, C_WSS_ contents were elevated in all groups, peaking in the Gly treatment, followed by the Cat, Glu, and CK treatments. Notably, by day 60, the C_WSS_ contents of all MRP-amended treatments had fallen below that of the CK, with the Glu treatment maintaining the lowest level throughout the subsequent stages. This indicated that MRPs effectively accelerate the overall degradation efficiency of C_WSS_, with Glu exerting the most prominent stimulatory effect. The MRPs enhance C_WSS_ degradation by stimulating might potentially modulate microbial metabolism via distinct pathways. As a readily degradable C source, Glu might directly supply abundant C skeletons and energy, continuously fueling microbial metabolic activity [17]; consequently, the degradation and utilization efficiency of C_WSS_ in the Glu treatment was significantly higher than that of the CK. This proposed mechanism was inferred from bulk chemical parameters and had not been verified by direct quantification of microbial biomass or activity. Meanwhile, the Cat treatment was postulated to potentially activate putative phenol-degrading bacteria, and the Gly treatment provided essential supplementary organic N [17]; furthermore, the superior C_WSS_ degradation observed in the Glu treatment logically corresponded with its significant promoting effect on microbially derived DOM previously noted in the incubation system. This alignment robustly corroborates the positive regulatory role of Glu on microbial metabolic activity.

**Fig 7:**
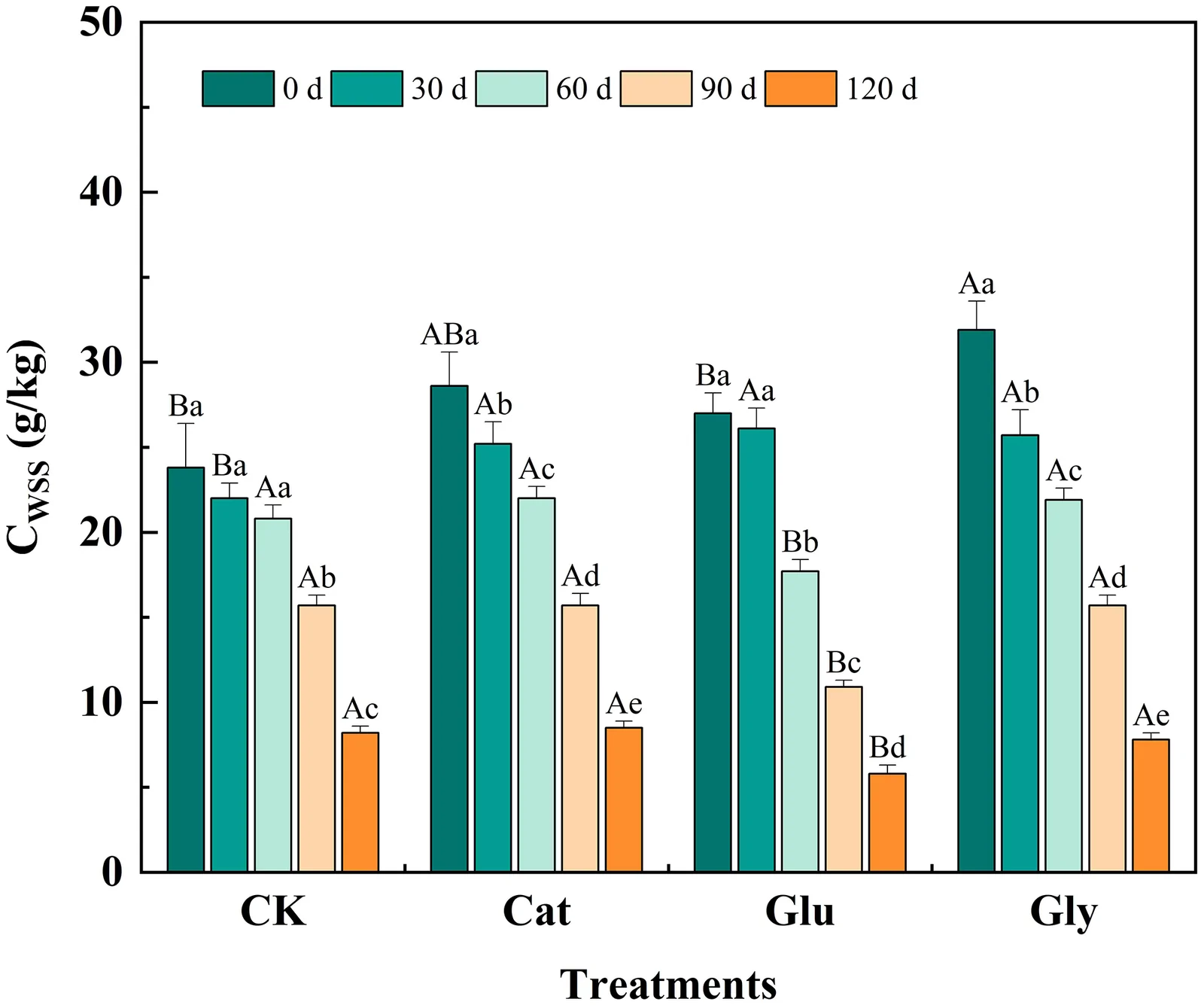
Dynamic changes in C_WSS_ during simulated mesophilic co-composting incubation. Note: Uppercase letters denote statistically significant differences among treatments at the same composting stage, whereas different lowercase letters denote significant differences across composting days within the same treatment.

### 3.8. Effects of MRPs addition on C_HE_ during simulated mesophilic co‑composting incubation

In contrast to the continuous depletion of C_WSS_, the C_HE_ progressively accumulated across all treatments throughout the incubation process. This accumulation indicated the persistent occurrence of the Maillard reaction and the robust progression of microbial-mediated humification (Fig 8). The C_HE_ contents in the CK, Gly, and Cat treatments exhibited a stable upward trend, whereas the Glu treatment displayed an initial decrease followed by a subsequent increase. By day 120, the Glu treatment achieved the highest C_HE_ content (57.7 g/kg); however, due to its initial depletion phase, its net relative increase from the initial stage was only 16.6%. In terms of relative accumulation, the Cat and Gly treatments exhibited higher increases of 32.0% and 26.8%, respectively, while the CK showed an 18.3% increase. These distinct temporal trajectories clearly demonstrated the differential enrichment effects of the various MRPs on C_HE_ accumulation. The dynamic variations in C_HE_ were fundamentally governed by the distinct action modes of the specific MRPs. The early C_HE_ decrease observed in the Glu treatment was likely driven by the preferential microbial utilization of small-molecule reducing sugars for rapid proliferation, a process that actively competed for resources and temporarily inhibited C_HE_ synthesis [29]. Subsequently, during the middle and late stages, the Glu treatment might supply abundant C skeletons for the Maillard reaction, thereby was proposed to drive a continuous C_HE_ resurgence [17]. The Cat treatment was hypothesized to accelerate C_HE_ synthesis by promoting the hypothesized oxidative coupling of HS precursors via its reactive phenolic –OH groups [30]. Meanwhile, the Gly treatment effectively supplemented essential N for the Maillard condensation reaction, facilitating precursor conjugation and ensuring steady C_HE_ enrichment [20].

**Fig 8:**
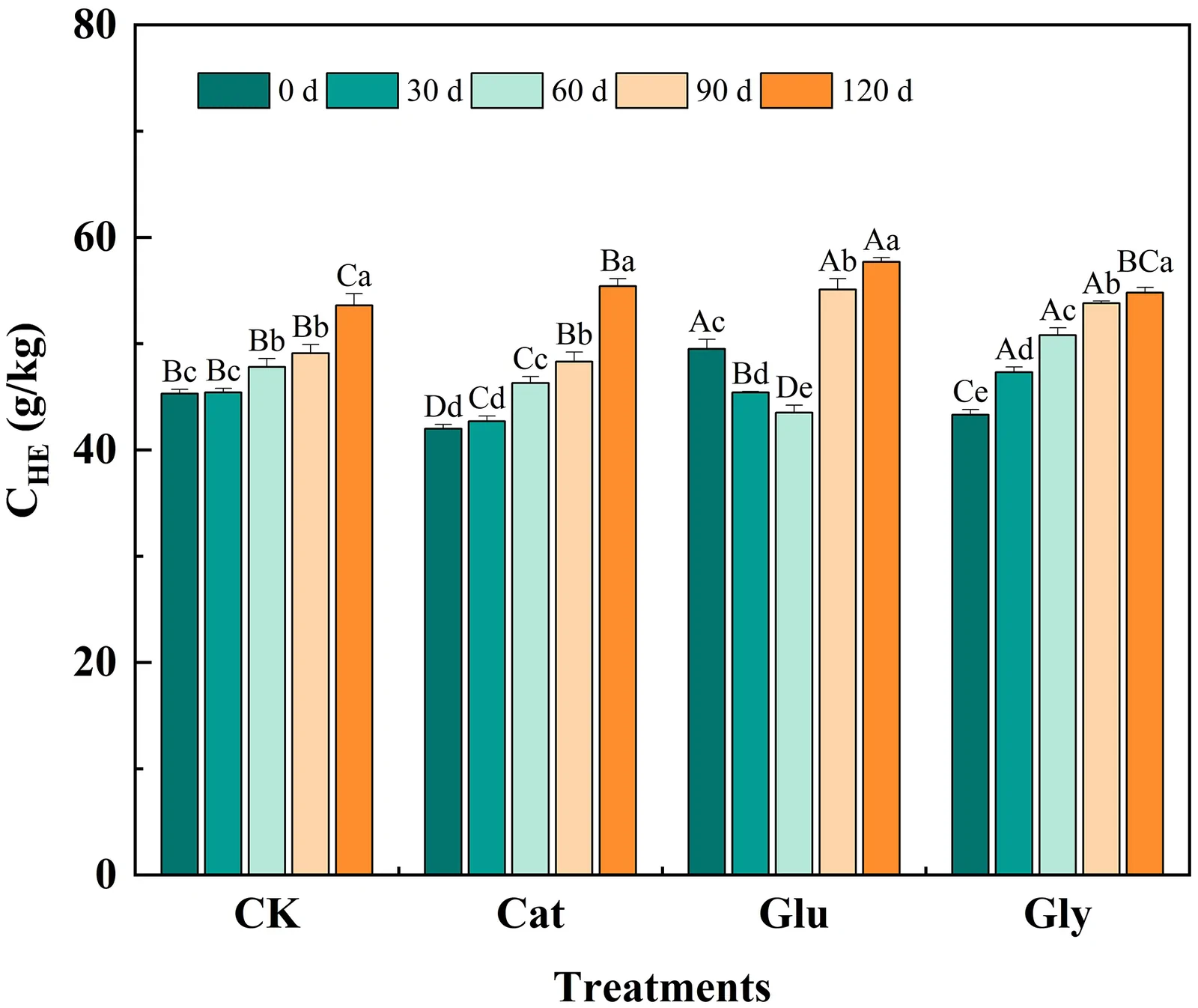
Dynamic changes in C_HE_ during simulated mesophilic co-composting incubation. Note: Uppercase letters denote statistically significant differences among treatments at the same composting stage, whereas different lowercase letters denote significant differences across composting days within the same treatment.

### 3.9. Effects of MRPs addition on C_HA_ during simulated mesophilic co‑composting incubation

The C_HA_ exhibited significant inter-treatment variation, which indicated a dynamic equilibrium between the microbial degradation of macromolecular HA and HS precursors (Fig 9). The CK and Cat treatments exhibited an initial decrease followed by a subsequent increase in C_HA_, whereas the Glu and Gly treatments displayed a continuous decline. By day 120, the resurgent C_HA_ in the CK and Cat treatments was attributed to the robust condensation of FA into HA; the Cat treatment further promoted this process via active aromatic cross-linking [31]. The excessive C_HA_ rebound observed in the CK was likely caused by the spontaneous condensation of FA into HA, driven by the severe depletion of labile C. In contrast, Glu potentially stimulated specific aromatic compound-degrading microbes, thereby persistently suppressing bulk HA accumulation. Meanwhile, Gly facilitated the polymerization of amino acids with phenolic compounds to form stable HS and enhanced overall HS stability through metal ion chelation, ultimately maintaining a consistently low concentration of C_HA_ [32–34].

**Fig 9:**
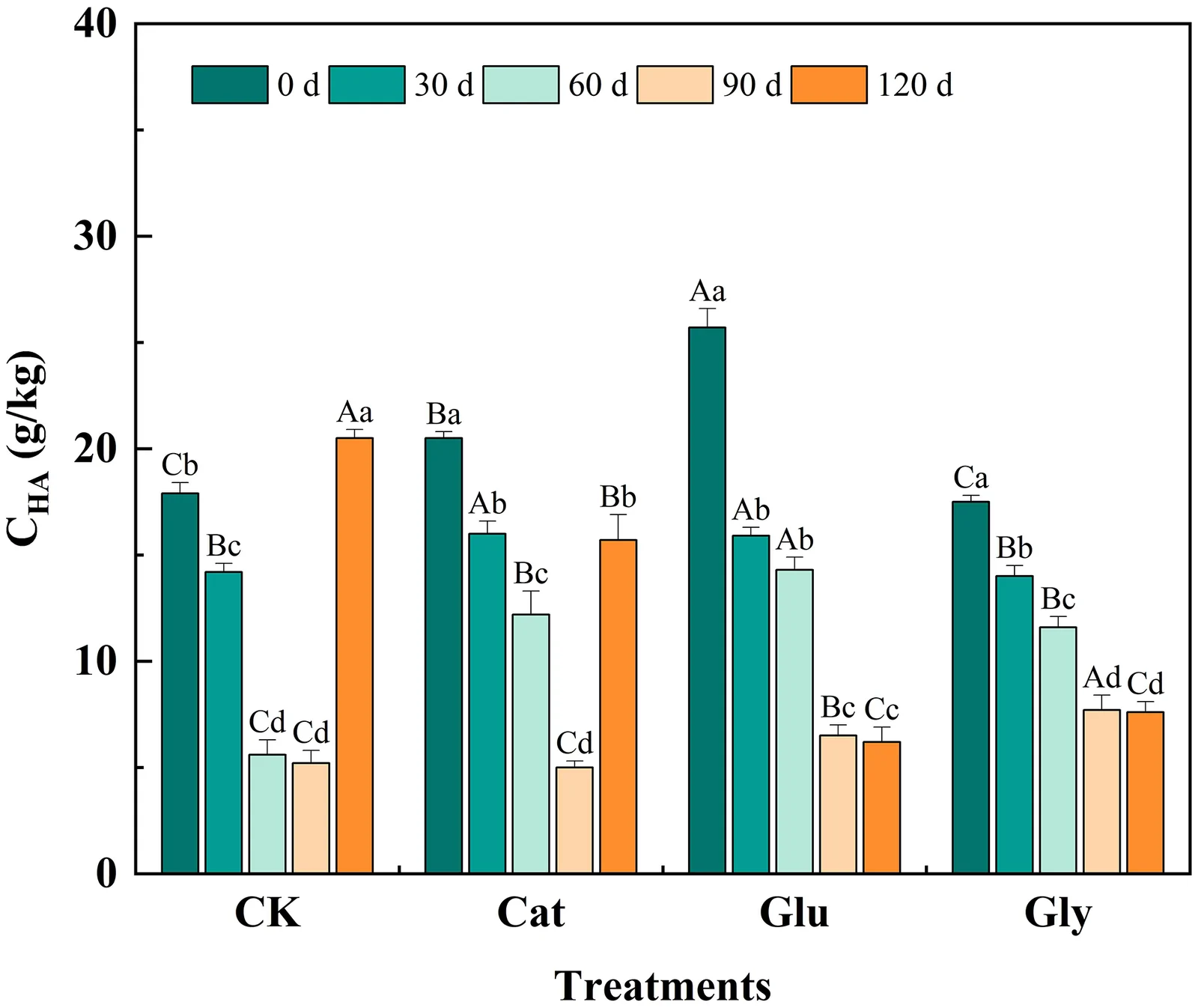
Dynamic changes in C_HA_ during simulated mesophilic co-composting incubation. Note: Uppercase letters denote statistically significant differences among treatments at the same composting stage, whereas different lowercase letters denote significant differences across composting days within the same treatment.

### 3.10. Effects of MRPs addition on DP during simulated mesophilic co‑composting incubation

The DP, a critical parameter reflecting HS polymerization and humification, was positively correlated with C_HA_, and its temporal dynamics were highly consistent with the C_HA_ trends across all treatments [13] (Fig 10). By day 120, the DP of the CK rebounded to its initial level. The subsequent DP rebound in the CK reflected the intrinsic self-recovery capacity of HS polymerization under unamended mesophilic decomposition conditions. However, the DP obtained from the three different processing methods that incorporated MRP remained at a relatively low level, which confirmed their sustained, type-specific regulatory effects on HS polymerization. Importantly, this intervention effectively drove the humification trajectory away from natural macromolecular HA formation, favoring the accumulation of lower-molecular-weight FA and highly active HS components [35]. Compared to highly polymerized, recalcitrant macromolecular HA, these low-molecular-weight and highly active HS components possessed superior water solubility, a higher density of O-containing functional groups, and stronger mobility. In practical agricultural applications, they could more efficiently chelate soil trace elements, enhance nutrient bioavailability, and be more easily absorbed by plant root systems to stimulate crop metabolism.

**Fig 10:**
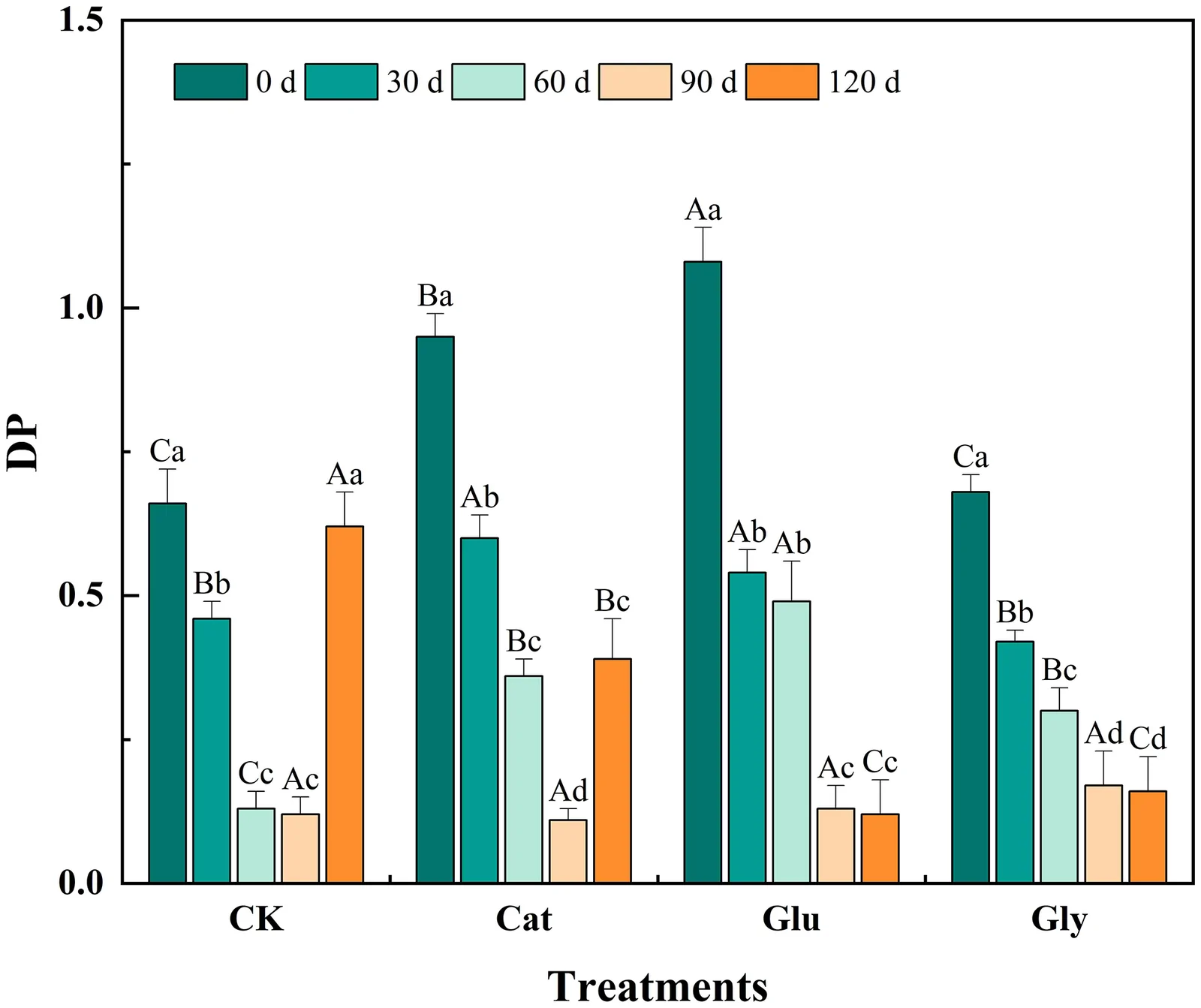
Dynamic changes in DP values during simulated mesophilic co-composting incubation. Note: Uppercase letters denote statistically significant differences among treatments at the same composting stage, whereas different lowercase letters denote significant differences across composting days within the same treatment.

### 3.11. Effects of MRPs addition on C_Hu_ during simulated mesophilic co-composting incubation

Hu, as the most recalcitrant and stable component of HS, directly reflected the regulatory efficacy of MRPs on C stabilization during the mesophilic humification process [36]. As depicted in Fig 11, the C_Hu_ in all MRP-amended treatments experienced rapid depletion during the initial phase (0–30 days). The reduction amplitudes for the MRP treatments were substantially greater than that of the CK. However, by day 120, all MRP treatments ultimately achieved higher final C_Hu_ levels than the CK, which demonstrated that the addition of MRPs fundamentally promoted long-term C stabilization during simulated mesophilic co-composting. The rapid initial depletion of C_Hu_ in the MRP treatments was likely driven by the intense stimulation of microbial metabolism. During this hyperactive phase, microorganisms potentially decomposed not only the readily available C_WSS_ but also the more labile organic fractions loosely bound to the Hu matrix to fulfill their elevated growth and metabolic demands. Conversely, the subsequent C_Hu_ accumulation during the later stages was synergistically driven by sustained microbial metabolism and the ongoing Maillard reaction. Specifically, the extensive OM degradation during the early and middle stages generated a massive pool of HS precursors; the presence of MRPs subsequently facilitated the progressive conjugation of these precursors with the Hu matrix, thereby actively driving the conversion of labile C into highly stable C_Hu_ and achieving effective C immobilization.

**Fig 11:**
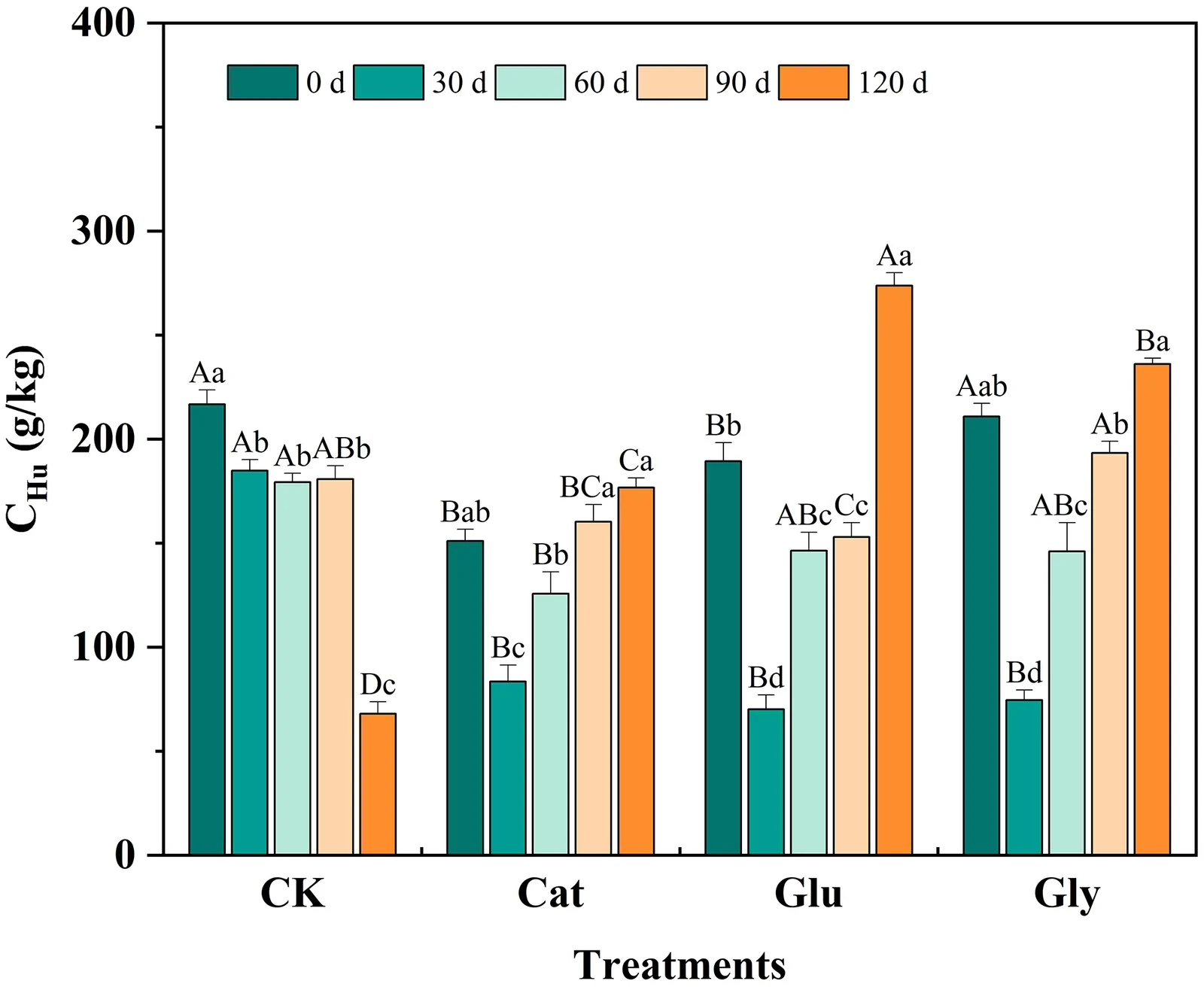
Dynamic changes in C_Hu_ values during simulated mesophilic co-composting incubation. Note: Uppercase letters denote statistically significant differences among treatments at the same composting stage, whereas different lowercase letters denote significant differences across composting days within the same treatment.

### 3.12. Effects of MRPs addition on atomic molar ratios of HA

All treatments exhibited a continuous decrease in the C/N ratio of HA throughout the 120-day mesophilic incubation period (Table 1). Among these, the Gly treatment achieved the greatest reduction of 17.1%, followed by the Glu treatment with a 14.3% decrease. In contrast, the CK experienced a 9.5% reduction, and the Cat treatment recorded the smallest decrease at 8.8%. The universal decline in the C/N ratio across all treatments suggests enhanced microbial N immobilization and advanced molecular condensation, both of which were essential for HA maturation [37]. The Gly treatment delivered the most substantial C/N reduction by supplementing organic N and inducing relative C limitation; this likely accelerated the mineralization of labile C and promoted the formation of N-containing aromatic heterocycles via the Maillard reaction [20]. Conversely, Glu reduced the C/N ratio by providing abundant C skeletons that facilitated C–N coupling during non-enzymatic polymerization [37]. The Cat treatment, however, maintained a relatively stable C/N ratio, potentially because its specific phenolic structure stabilized the HA aromatic core through oxidative coupling, thereby restraining excessive C mineralization [38]. The O/C ratio of HA increased universally across all treatments during the incubation process. The CK exhibited the most pronounced increase in the O/C ratio, whereas the Cat treatment demonstrated the mildest increase. The Glu and Gly treatments displayed intermediate increases of 6.8% and 10.5%, respectively. This increase in the O/C ratio demonstrated the continuous oxidation of HA molecules, a process highly conducive to enhancing their hydrophilicity and functional reactivity [39]. In contrast, the Cat treatment likely alleviates over-oxidation through the putative free radical scavenging properties of its phenolic hydroxyl groups, thereby slowing the oxidation rate of HA. Concurrently, Glu and Gly might achieve moderate, regulated oxidation through putative glycosylation interactions and modified decarboxylation pathways, respectively. The H/C ratio served as a critical indicator for evaluating the degree of HA aromatization [40]. Across all treatments, this ratio declined significantly from day 0 to day 120. The reduction in the Gly treatment was the most drastic at 36.8%, far exceeding the other groups. The CK exhibited a 21.3% decline, followed by the Glu (17.0%) and Cat (14.7%) treatments. The consistent decrease in the H/C ratio verified the enhanced aromatization of HA throughout the mesophilic humification process, and the significant inter-treatment variations further indicated that the specific MRPs could distinctly regulate this aromatization trajectory. Specifically, Gly was hypothesized to induce the most intense aromatization, likely by promoting the hypothesized massive formation of N-containing aromatic heterocycles via the Maillard reaction [40]. In contrast, Glu and Cat appear to protect the aliphatic moieties within the HA structure, facilitating the development of a balanced aliphatic-aromatic architecture that strongly supports the functional stability of HA.

**Table 1:** Atomic molar ratios of HA from different treatments at the initial and final stages of simulated mesophilic PM-SMS co-composting incubation.

Treatments	Composting duration (d)	C/N ratio	O/C ratio	H/C ratio
CK	0	28.3 ± 0.8	0.56 ± 0.02	1.55 ± 0.17
	120	25.6 ± 0.7	0.63 ± 0.06	1.22 ± 0.14
Cat	0	13.6 ± 0.3	0.69 ± 0.04	1.36 ± 0.11
	120	12.4 ± 0.4	0.72 ± 0.05	1.16 ± 0.10
Glu	0	16.1 ± 0.2	0.74 ± 0.04	1.41 ± 0.11
	120	13.8 ± 0.6	0.79 ± 0.08	1.17 ± 0.07
Gly	0	15.2 ± 0.4	0.76 ± 0.06	1.36 ± 0.09
	120	12.6 ± 0.5	0.84 ± 0.09	0.86 ± 0.05

### 3.13. Effects of MRPs addition on FTIR spectral characteristics of HA

As depicted in Fig 12 and S2 Table, the absorption band at 3401–3433 cm^-1^ corresponds to O–H and N–H stretching vibrations. This band reflected both inter- and intramolecular H-bonding interactions, alongside the relative abundance of hydrophilic –OH and –NH_2_ functional groups [41]. On the 0th day, the peak values of the treatment group with MRP were all higher than those of CK. It is notable that the Glu treatment showed the highest initial intensity at the spectral band (48.1%), which was mainly attributed to the abundant –OH groups inherent in the added Glu molecules. Over the 120-day incubation period, all treatments exhibited a continuous decline in the intensity of this peak. The progressive weakening of the O–H/N–H stretching vibrations indicated a reduction in hydrophilic groups, a process fundamentally driven by the extensive condensation of HA molecules during humification. The Cat treatment likely retained the highest hydrophilicity because its specific phenolic structure potentially enhanced H-bonding interactions between HA molecules through oxidative coupling, thereby mitigating the extensive loss of these hydrophilic groups. In contrast, the Gly treatment strongly promoted the condensation of HA molecules, leading to the most substantial depletion of hydrophilic groups to the lowest level; this aligned perfectly with its robust aromatization capability, as previously corroborated by its remarkably low H/C ratio. Furthermore, the absorption band at 2921–2968 cm^-1^ corresponds to the asymmetric stretching vibrations of aliphatic C–H bonds, while the band at 2847–2873 cm^-1^ is ascribed to their symmetric stretching vibrations [42]. Both aliphatic C–H peaks decreased steadily from day 0 to day 120 across all treatments, which confirmed the continuous microbial degradation of aliphatic side-chains in HA during mesophilic humification [43]. Notably, at maturity, the Cat treatment retained the highest total intensity for these aliphatic peaks among the MRP treatments, whereas the Glu treatment exhibited the most severe overall depletion. The superior retention of aliphatic C–H signals in the Cat treatment indicated that it effectively protected aliphatic moieties from excessive degradation. Conversely, the Glu and Gly treatments likely promoted the active degradation of aliphatic structures to supply essential C sources for the continuous synthesis of aromatic rings, thereby fundamentally accelerating the overall aromatization of HA. The absorption band at 1564–1596 cm^-1^ primarily originates from aromatic ring C = C stretching and the amide II band, representing a coupled vibration of N–H bending and C–N stretching [41]. Over the 120-day period, the relative intensity of this peak increased across all treatments. Notably, the Gly treatment reached the highest final value (21.0%), followed by Cat (19.8%) and Glu (18.7%), all of which significantly surpassed the CK (17.9%). This intensified absorption indicated that the addition of MRPs strongly enhanced the formation of aromatic structures and amide groups within HA. Coupled with its lowest final H/C ratio, the Gly treatment demonstrated the most potent ability to promote aromatic structure formation. This robust aromatization was attributed to the Gly-induced Maillard reaction, which facilitated the massive formation of N-containing aromatic heterocycles [40]. Furthermore, the prominent formation of amide groups suggested that MRPs likely promoted the active coupling of N-containing compounds with aromatic cores, a structural evolution highly conducive to improving both the stability and functional activity of HA molecules. The band at 1407–1461 cm^-1^ was assigned to aliphatic C–H bending vibrations and the asymmetric stretching vibrations of free carboxyl (COO^–^) groups [41]. While this peak’s intensity exhibited a slight decrease in all groups during the incubation process, the MRP-amended treatments maintained substantially lower overall intensities compared to the CK throughout the entire 120-day period. This persistently low abundance of free COO^–^ groups indicated that MRPs potentially facilitated the conversion of free COO^–^ groups into amide groups via the Maillard condensation reaction, thereby fundamentally reducing the residual free COO^–^ content [32]. Simultaneously, the concurrent reduction in aliphatic C–H bending vibrations further corroborates the microbial degradation of aliphatic side chains in HA, firmly aligning with the earlier observations of the aliphatic C–H stretching peaks. The peak at 1033–1052 cm^-1^ corresponds to C–O stretching vibrations, primarily reflecting the abundance of O-containing functional groups associated with polysaccharide-like structures in HA [41]. Its intensity slightly decreased across all groups. However, the Glu treatment exhibited the smallest overall reduction among the MRP treatments, thereby retaining a robust carbohydrate-derived structural signature in HA by day 120. This preservation was fundamentally due to the fact that Glu, as a highly labile monosaccharide precursor, actively supplemented the carbohydrate pool during the mesophilic incubation process. In contrast, the more pronounced reductions observed in the Gly and Cat treatments suggested that these precursors partially stimulated the degradation of intrinsic polysaccharides to provide necessary C sources for aromatic ring synthesis. Consequently, hypothesized glycosylation interactions between Glu and HA molecules were proposed to mitigate the microbial degradation of intrinsic polysaccharide structures. The aliphatic C/aromatic C ratio is calculated as (a + b + c)/d from FTIR relative intensities [44]. From day 0 to day 120, this ratio decreased continuously across all treatments, which reflected the microbial degradation of aliphatic side chains and the concurrent condensation of stable aromatic cores [45]. By maturity, all MRP-amended treatments exhibited substantially lower (a + b + c)/d ratios (0.75–0.84) compared to the CK (1.10). Notably, the Cat and Gly treatments achieved the lowest final value (0.75), which perfectly corroborated their pronounced reductions in the H/C ratio. This robust aromatization is putatively driven by the Gly-induced Maillard reaction and Cat-mediated oxidative coupling [30,40]. Meanwhile, the slightly higher ratio in the Glu treatment (0.84) indicated a balanced retention of carbohydrate-derived aliphatic moieties.

**Fig 12:**
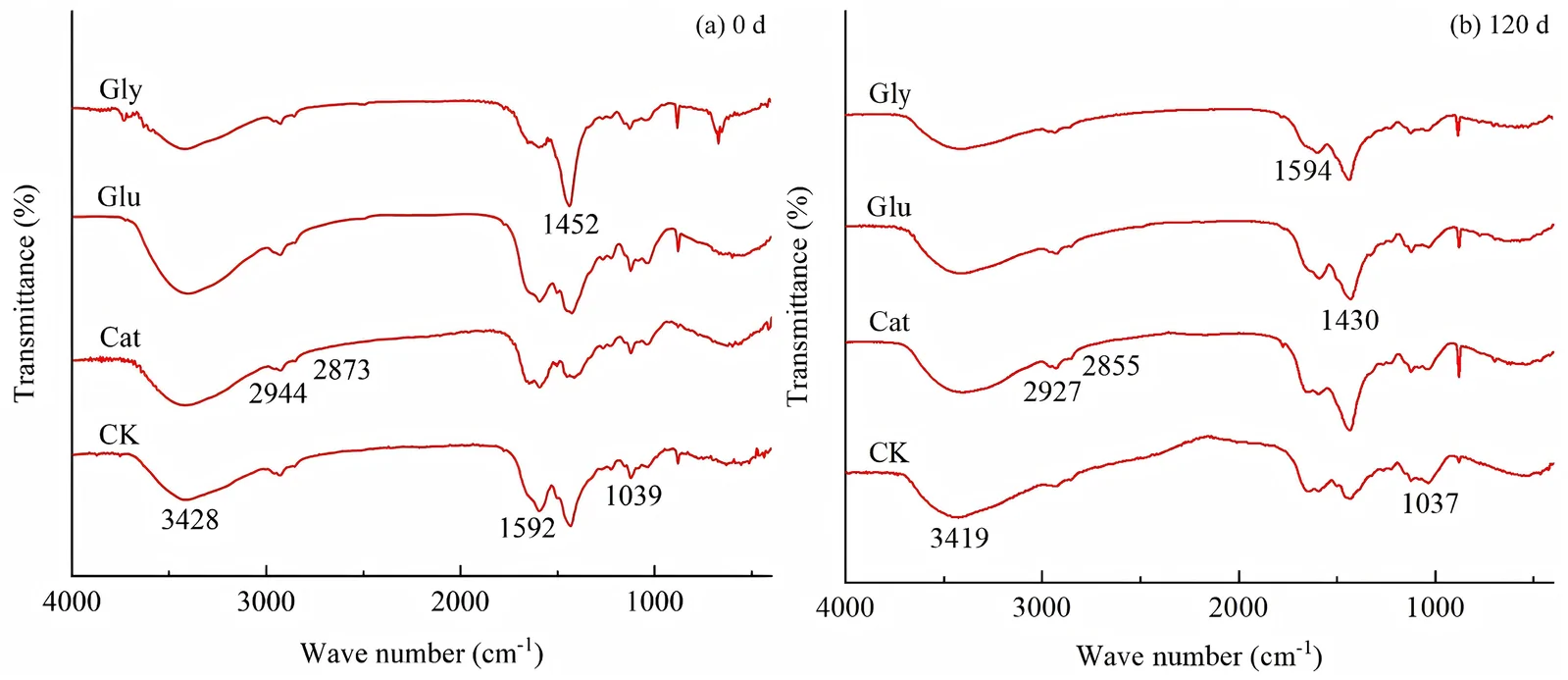
FTIR spectra of HA obtained during the initial (a) and final (b) stages of simulated mesophilic PM‑SMS co‑composting incubation.

### 3.14. Comprehensive analysis

As shown in Fig 13, PCA was performed to systematically quantify the regulatory effects of MRPs on HS composition and chemical properties of the simulated mesophilic PM‑SMS co‑composting system over the 120-day composting period. Three PCs were extracted, with PC1 accounting for 55.472% of the total variation, PC2 for 27.915%, and PC3 for 16.613% (S3 Table). The three PCs collectively captured all variation information of humification quality parameters and formed a reliable comprehensive evaluation system, with the PCA score plot showing a clear separation of MRP treatments from the CK in the multidimensional indicator space, indicative of the distinct regulatory roles of different MRPs. Based on the loading coefficients of humification quality parameters on each PC, the ecological implications of the three PCs were clearly defined. PC1 was identified as the core component reflecting the mesophilic humification process and basic nutritional quality of the system. PC2 represented the dynamics of nutrient transformation and HS composition variation during the incubation period, which was mainly driven by variables related to HS structural evolution. PC3 was the PC governing salt dynamics and nutrient balance maintenance. Based on the eigenvector matrix in S4 Table, linear equations for calculating the scores of the three PCs were established (Equations 1–3).


$$\begin{array}{c} \begin{array}{c} F_{1}=0.082\times Total\text{\textasciitilde{}}nutrients+0.082\times OM-0.085\times C_{WSS}+0.117\times \;C_{HE}-0.128\times C_{HA}+0.137\\ \times \;C_{Hu}-0.04\times EC+0.117\times Gl-0.133\times DP-0.126\times C/N\text{\textasciitilde{}}ratio+0.124\\ \times \;O/C\text{\textasciitilde{}}ratio-0.059\times H/C\text{\textasciitilde{}}ratio-0.027\times [\text{(a+b+c)/d}] \end{array} \end{array}$$ (1)

$$\begin{array}{c} \begin{array}{c} F_{2}=0.181\times Total\text{\textasciitilde{}}nutrients+0.181\times OM-0.187\times C_{WSS}+0.147\times \;C_{HE}-0.002\times C_{HA}+0.007\\ \times \;C_{Hu}-0.102\times EC+0.128\times Gl-0.013\times DP-0.086\times C/N\text{\textasciitilde{}}ratio+0.091\\ \times \;O/C\text{\textasciitilde{}}ratio-0.217\times H/C\text{\textasciitilde{}}ratio-0.254\times [\text{(a+b+c)/d}] \end{array} \end{array}$$ (2)

$$\begin{array}{c} \begin{array}{c} F3=0.218\times Total\text{\textasciitilde{}}nutrients+0.218\times OM-0.189\times C_{WSS}+0.036\times \;C_{HE}-0.174\times C_{HA}+0.064\\ \times \;C_{Hu}-0.399\times EC+0.128\times Gl-0.128\times DP-0.qw8\times C/N\text{\textasciitilde{}}ratio+0.145\\ \times \;O/C\text{\textasciitilde{}}ratio-0.206\times H/C\text{\textasciitilde{}}ratio-0.158\times [\text{(a+b+c)/d}] \end{array} \end{array}$$ (3)

**Fig 13:**
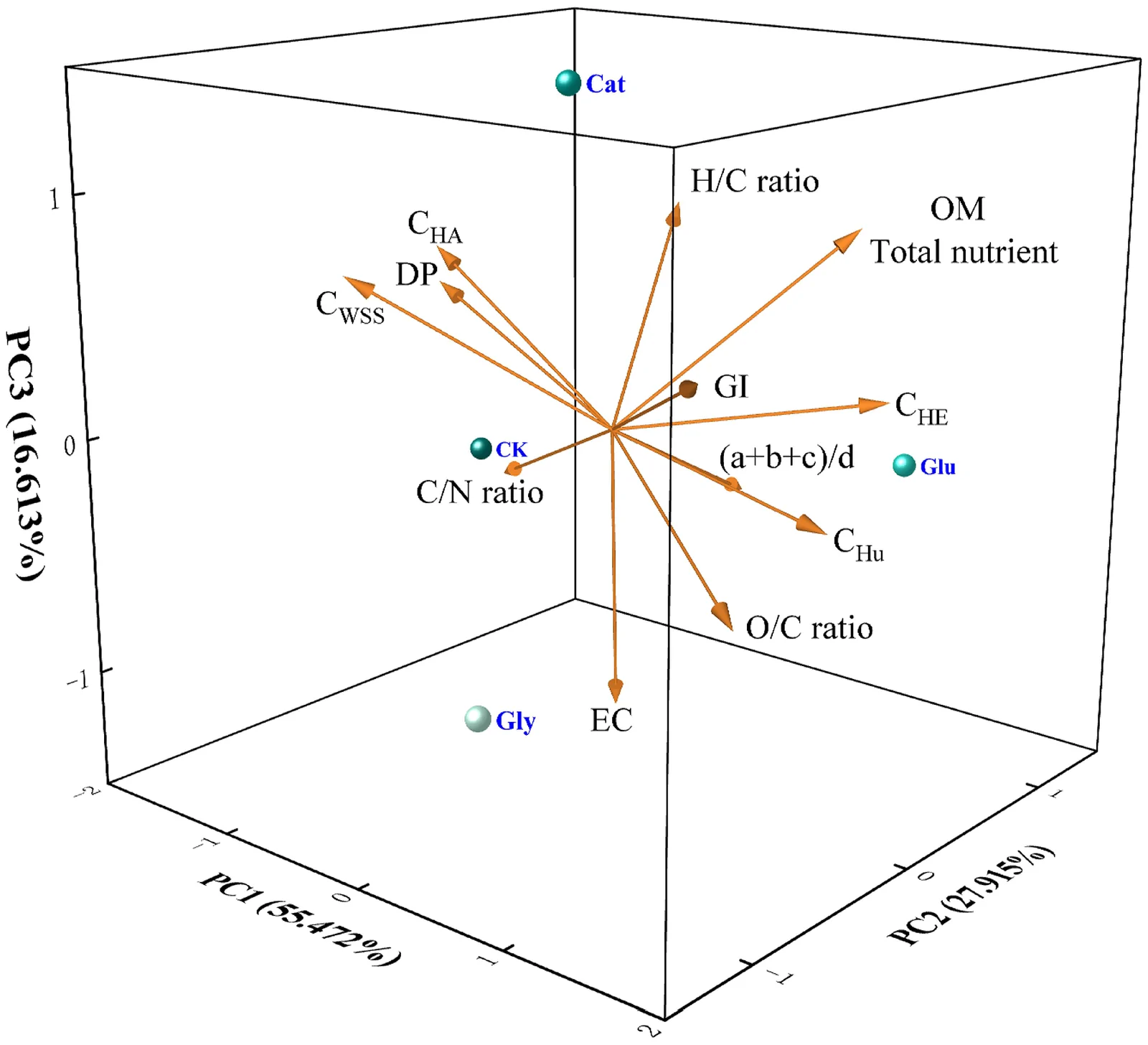
Effects of different MRPs on simulated mesophilic co-composting of SMS and PM: Insights from PCA. Note: PCA composite scores and treatment rankings across different MRP amendment groups. The composite score was calculated using the variance contribution ratio of each extracted principal component as objective weights. The derived ranking is valid only for this weighting strategy; without sensitivity validation, it represents a preliminary indicative finding rather than a robust universal conclusion.

The individual scores (F_1_ for PC1, F_2_ for PC2, F_3_ for PC3) and comprehensive scores of each treatment at the mature stage of the mesophilic incubation (120 d) **were** calculated based on the above equations and the weighted summation method (weight = individual contribution rate of PC/100), and the results are shown in S5 Table. The comprehensive score was calculated as Equation 4, which integrated the information of all three PCs to realize the quantitative ranking of comprehensive humification quality under different treatments.


$$\begin{array}{c} Comprehensive\text{\textasciitilde{}}score=0.555\times F_{1}+0.279\times F_{2}+0.166\times F_{3} \end{array}$$ (4)

The comprehensive scores reflect the overall effects of different MRPs on improving humification quality in the simulated SMS‑PM mesophilic co‑composting system, with the order being Glu (0.772)> Cat (0.192)> Gly (−0.215)> CK (−0.749). Notably, all MRP addition treatments had higher comprehensive scores than CK, which confirmed that MRPs could effectively improve the comprehensive quality of mesophilic humification products, and the regulatory effects of different MRPs were different due to their distinct action mechanisms.

PC1, as the dominant component, was closely associated with humification indicators and basic nutritional properties, representing the core dimension of mesophilic humification and fundamental product quality. PC2 mainly reflected nutrient transformation and HS components, while PC3 was dominated by salinity regulation and nutrient balance maintenance. Glu achieved the highest scores in PC1 and PC2, demonstrating its superior comprehensive regulatory performance. As a reducing sugar precursor, Glu continuously supplied available C sources to synergistically drive the Maillard reaction and enhance microbial metabolism, thereby synchronously optimizing HS formation, nutrient enrichment, and agronomic safety, which made it the most effective MRP for overall humification quality improvement under mesophilic conditions. Cat exhibited the optimal performance in PC3, owing to its unique phenolic structure that activated specific functional microorganisms, accelerated soluble salt complexation and leaching, and effectively alleviated salinity stress and residual phytotoxicity, highlighting its advantages in humification product safety regulation. Gly primarily improved humification maturity by optimizing the C/N ratio and promoting C–N metabolic balance, but its overall regulatory effect was weaker than Glu and Cat due to C limitation in the system. The composite comprehensive score calculated using principal-component variance-contribution ratios as objective weighting coefficients gave the treatment order Glu > Cat > Gly > CK, confirming that all MRP amendments enhanced the comprehensive quality of humification products in the simulated PM-SMS mesophilic co-composting system compared with the CK. The discrete distribution of treatments in the PCA score plot further revealed the functional differentiation of MRPs: Glu mediated a comprehensive humification-nutrient optimization pathway, Cat represented a salinity-toxicity control pathway, and Gly acted as a maturity-promoting pathway.

### 3.15. Total effects and dominant driver identification

To systematically elucidate the causal relationships and quantify the direct and indirect effects of key factors on final humification product quality, a SEM was constructed (Fig 14). The total standardized effect analysis provided correlative statistical evidence consistent with our hypothesized causal framework, rather than conclusive biological proof that the addition of MRPs as the crucial exogenous driver shaped the overall simulated mesophilic co-composting system. Specifically, MRPs exerted a significant direct positive effect (0.364) on final humification product quality by continuously fueling non-enzymatic browning processes and the synthesis of HS. Beyond the direct impact, MRPs profoundly enhanced product quality through vital indirect pathways, primarily by synergistically promoting the humification process and optimizing the structural evolution of HS. Notably, the humification process exhibited a significant negative effect on HS structure (path coefficient = −0.163). This phenomenon was mechanistically consistent with the transient decline in HS polymerization observed during the mid‑incubation stage, which had been driven by the preferential microbial degradation of labile HS components and unstable high-molecular-weight HA before their eventual condensation. By quantifying the causal relationships among these latent variables, the SEM corroborated the trends identified in PCA and offered statistical support for our mechanistic hypothesis. It had structurally verified that Glu acted as the most potent MRP, manifesting the strongest regulatory effect by synchronizing the Maillard reaction with possibly enhanced microbial metabolism. In contrast, Cat and Gly governed humification product quality through more targeted, yet functionally narrower pathways—specifically by alleviating salinity stress and accelerating humification maturation, respectively.

**Fig 14:**
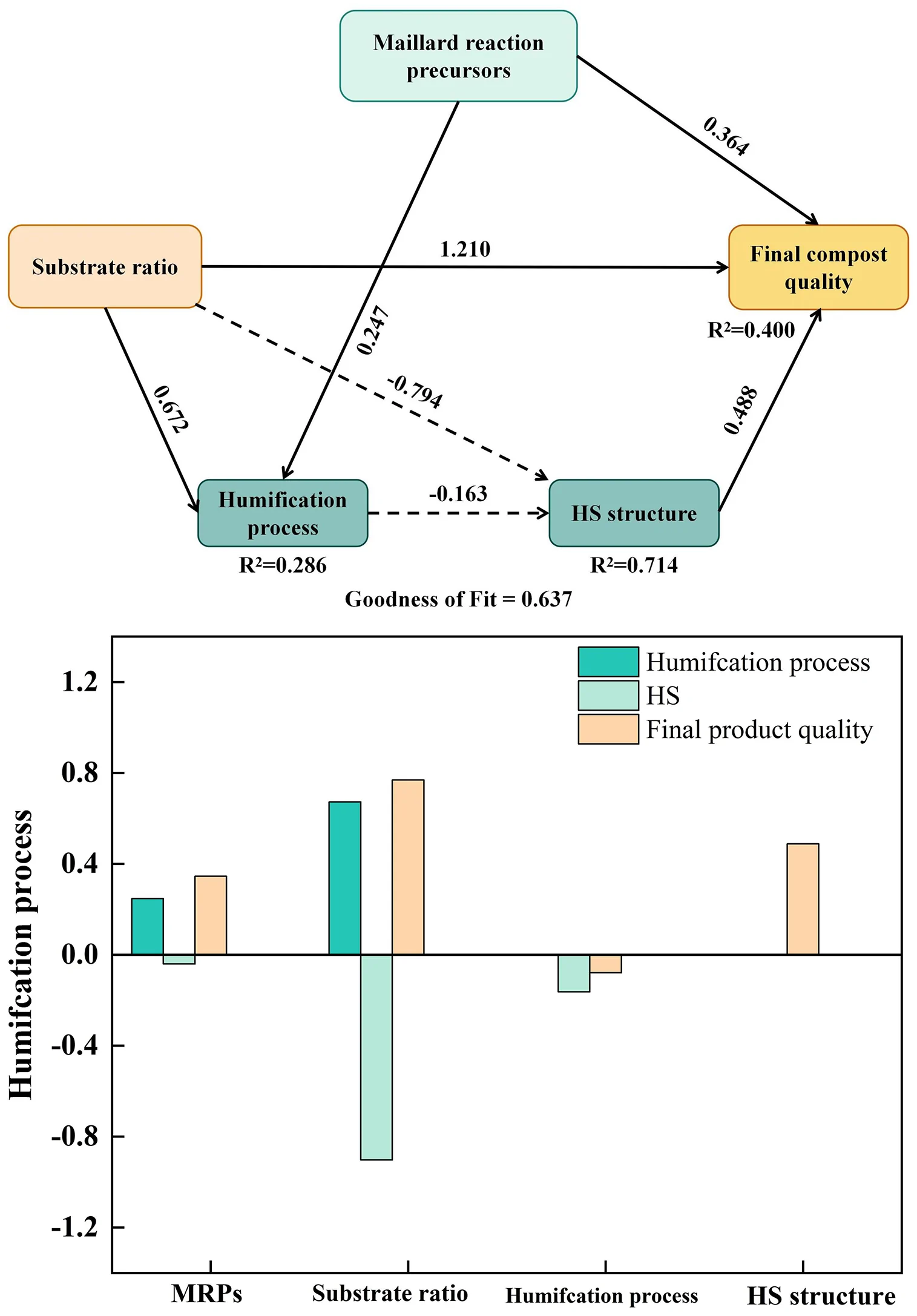
Structural equation model (a) and total standardized effects (b) of key factors regulating simulated mesophilic PM‑SMS co‑composting product quality. Note: Solid arrows indicate positive effects, dashed arrows indicate negative effects. Values on arrows are standardized path coefficients. All coefficients are significant at *p* < 0.05. R^2^ values represent the proportion of variance explained for each latent variable. Total effects = direct + indirect effects.

### 3.16. Linear mixed‑effects model

Linear mixed‑effects models were adopted for longitudinal repeated‑measures data to avoid violation of independence assumption, where each reactor was repeatedly subsampled across 0, 30, 60, 90 and 120 days. MRP treatment and incubation time were fixed factors, and reactor ID was specified as random intercept. Mixed‑effects outputs quantifying main and interactive effects of MRP amendment and incubation time on core humification‑related physicochemical parameters are summarized in Table 2. For total nutrient indicators, MRP addition exerted highly significant main effects (*p* < 0.01) on TP, TK, OM, and total nutrients, whereas its main effect on TN was non-significant (*p* ≥ 0.05). Incubation time produced highly significant main effects on TP, TK, OM, and total nutrients (*p* < 0.01), but showed no significant main effect on TN. Significant interactive effects between MRPs and incubation time were observed for TP, TK, OM, and total nutrients (*p* < 0.05 or *p* < 0.01); the interaction term for TN became non‑significant under mixed‑effects modelling. These interactions indicated that the temporal variation of these nutrient properties was strongly dependent on the type of MRP, meaning that the differences among treatments gradually diverged or converged as the incubation process proceeded. The non-significant main effect of MRPs on TN implied that MRP input could not sustainably alter the net total-N pool of the matrix, even for Gly, which provided extra N-containing substrates. With respect to maturity indices, both MRPs and incubation time exerted highly significant main effects on GI (*p* < 0.01), and their interaction was also highly significant (*p* < 0.01). By comparison, main effects and interactive effects for EC were all non-significant (*p* ≥ 0.05). This suggested that MRP regulation of humification maturity was time-dependent, while MRP addition barely changed the overall salinity level throughout the whole incubation period. For HS fractions, MRPs generated significant or highly significant main effects on C_WSS_ and C_HA_ (*p* < 0.05) or (*p* < 0.01), but the main effect on C_HE_ was ns. Incubation time imposed highly significant main effects on C_WSS_, C_HA_, and C_HE_ (*p* < 0.01). A highly significant MRP × Incubation time interaction was detected for C_HA_ (*p* < 0.01), whereas interactions for C_WSS_ and C_HE_ were NS. It could be inferred that MRPs mainly regulated the formation of the HA fraction rather than the easily‑extractable HS C pool, and such regulatory effect varied distinctly at different incubation stages. Regarding humification and fluorescence spectroscopic parameters, MRPs, and incubation time both exhibited highly significant main effects on C_Hu_ and DP (*p* < 0.01), accompanied by highly significant interactive effects (*p* < 0.01). Main and interactive effects for FI were non-significant (*p* ≥ 0.05). Incubation time showed a highly significant main effect on BIX (*p* < 0.01), and the MRP × Incubation time interaction was also highly significant, alongside a highly significant main effect of MRPs on BIX. Neither MRPs nor incubation time induced significant changes for HIX. The index (a + b + c)/d responded significantly to both main factors as well as their interaction (*p* < 0.05) or (*p* < 0.01). The absence of responses for FI and HIX demonstrated that these two fluorescence proxies were insensitive to MRP-driven humification transformation under constant temperature conditions and were not suitable for distinguishing treatment differences in the present system. MRP amendment produced highly significant main effects on C/N, O/C, and H/C ratios. Incubation time exerted highly significant main effects on C/N and H/C (*p* < 0.01), while its main effect on O/C was ns. Notably, none of these elemental stoichiometry parameters displayed significant MRP × incubation time interactive effects (*p* ≥ 0.05). This was a key observation: although MRPs reshaped the initial elemental composition of the incubation matrix, the subsequent temporal transformation trajectories of elemental moieties were consistent across all MRP treatments. MRPs altered the starting status of elemental features instead of modifying their transformation rates over the incubation period.

**Table 2:** Mixed‑effects model outputs of core humification‑related parameters.

Different indicators	MRPs (fixed effect)	Incubation time (fixed effect)	MRPs × Incubation time (fixed interaction)
TN	ns	ns	ns
TP	**	**	**
TK	**	**	**
OM	**	**	*
Total nutrients	**	**	*
GI	**	**	**
EC	ns	ns	ns
C_WSS_	*	**	ns
C_HA_	**	**	**
C_HE_	ns	**	ns
C_Hu_	**	**	**
DP	**	**	**
FI	ns	ns	ns
BIX	**	**	**
HIX	ns	ns	ns
(a + b + c)/d	**	**	*
C/N	**	**	ns
O/C	**	ns	ns
H/C	**	**	ns

Note: Reactor ID is set as random intercept to account for repeated destructive subsampling on identical reactors across sampling time points. ** indicates a highly significant difference (*p* < 0.01); * indicates a statistically significant difference (*p* < 0.05); ns denotes no statistically significant difference (*p* ≥ 0.05).

## 4. Study limitations

The present study mainly focused on physicochemical and spectroscopic changes of humification-related properties under different MRP amendments. Neither microbial community profiling nor enzyme‑activity measurements were conducted in this work. Accordingly, interpretations concerning activation of phenol-degrading bacteria, stimulation of microbial metabolic activity (particularly the proposed metabolic enhancement by glucose), shifts in microbial community composition, and phenolic-driven enhanced oxidative‑coupling processes are mechanistic speculations derived from chemical‑spectroscopic observations and existing literature, and cannot be directly verified by our current dataset. Future complementary experiments, including high-throughput sequencing and enzyme assays, are needed to validate these potential microbial-related mechanisms. Moreover, structural interpretation of HS in the present study is exclusively based on FTIR spectroscopy and elemental analysis. High-resolution complementary techniques, including solid-state ^13^C NMR and Py-GC/MS were not implemented. This experiment was performed under constant controlled laboratory mesophilic conditions to specifically isolate temperature-independent humification mechanisms, rather than reproducing the dynamic thermal regime of industrial full-scale composting. Variations in temperature, aeration, substrate heterogeneity, and other field-relevant parameters were not reproduced in the present setup. Further pilot or full-scale composting trials are needed to verify its practical applicability. In addition, gas‑emission monitoring for NH_3_ and CO_2_ was absent; thus, N loss and C mineralization dynamics could not be directly quantified in this work.

## 5. Conclusions

The application of MRPs significantly optimized the humification process and final product quality during the mesophilic incubation simulating PM and SMS co-composting. Based on indirect chemical and spectroscopic evidence, we inferred divergent potential regulatory pathways for each MRP under mesophilic conditions: Glu exerted the strongest comprehensive improvement, which we hypothesized was partially driven by enhanced microbial metabolic activity and led to maximized formation of stable HS; Cat effectively mitigated salinity stress and phytotoxicity, an outcome tentatively attributed to putative phenolic oxidative‑coupling reactions alongside preservation of aliphatic structures; and Gly most potently drove the aromatization and condensation of HA by providing essential N. PCA confirmed that all MRP amendments enhanced the comprehensive quality of mesophilic humification products (ranked as Glu > Cat > Gly > CK) and clearly delineated their functional differentiation: Glu mediated a comprehensive humification-nutrient optimization pathway, Cat represented a salinity-toxicity control pathway, and Gly acted as a maturity-promoting pathway. PLS-SEM provided exploratory statistical support for our hypothesized conceptual framework, suggesting MRPs may act as pivotal exogenous drivers that directly and indirectly enhance humification maturity and product quality by accelerating humification and optimizing the structural evolution of HS; This study also has several noteworthy experimental limitations. All incubations were performed under constant mesophilic controlled conditions, and only one mixing ratio of SMS‑PM was tested. Thus, our laboratory-scale observations could not be fully extrapolated to variable real-world field composting environments. From a practical perspective, our findings provide preliminary evidence for applying MRPs in large-scale aerobic composting. These results provide indirect insights into plausible precursor-specific humification pathways and offer a novel targeted strategy to modulate HS evolution for high-value recycling of agricultural organic wastes.

## Supporting information

S1 Table Fluorescence indices of DOM obtained at the initial and final stages of simulated mesophilic PM-SMS co-composting incubation. (DOCX)

S2 Table FTIR relative intensities (% of total area) for the HA as influenced by different MRPs. (DOCX)

S3 Table Eigenvalues of the three PCs, along with their individual and cumulative contribution rates. (DOCX)

S4 Table Load matrix and eigenvector matrix of each PC. (DOCX)

S5 Table Average score and total score of main components of adding different MRPs. (DOCX)

S1 File Graphical abstract. (JPG)

## References

[1] Gupta G, Maurya S, Singh D. Waste to wealth: sustainable approach of alchemizing mushroom SMS into bioenergy. Bioresour Technol. 2026;443:133767. doi: 10.1016/j.biortech.2025.133767 41386488

[2] Bouzidi A, Tlahig S, Yahia Y, Elfalleh W. Transforming date palm agro‑waste into organic compost: A review of sustainable farming systems. BioResources. 2026;21(2). doi: 10.15376/biores.21.2.bouzidi

[3] Cruz GSJ, Hermes PH, Miriam SV, López AM. J Soil Sci Plant Nutr. 2024;24:4898–917. doi: 10.1007/s42729-024-01880-0

[4] Li K, Han Q, Wang J, Dai D, Gao H, Gao J, et al. Optimizing glycine concentration to enhance gibbsite-catalyzed abiotic humification of catechol and glucose. PLoS One. 2025;20(11):e0335528. doi: 10.1371/journal.pone.0335528 41252411 PMC12626293

[5] Xu Z, Li R, Liu J, Xu X, Wang S, Gao F. Impact of ammonifying microorganisms on the stabilization and carbon conversion of cow manure and wheat husk co-composting. Chem Eng J. 2024;490:151626. doi: 10.1016/j.cej.2024.151626

[6] Wang S, Liu P, Wang M, Cui Y, Tuo Y, Zhao B. Evaluation of chemical properties and humification process during co-composting of spent mushroom substrate (Pleurotus ostreatus) and pig manure under different mass ratios. Int Biodeter Biodegr. 2024;193:105858.

[7] Jiao M, Yue F, Ren X, Zhan X, Xu W, Tang D K H. Enhanced humification attributed by the integration of fenton reagent and oxalic acid during a co-composting of swine manure and corn straw: impacts and the possible mechanisms. Chem Eng J. 2024;498:155579. doi: 10.1016/j.cej.2024.155579

[8] Zhu N, Zhu Y, Liang D, Li B, Jin H, Dong Y. Enhanced turnover of phenolic precursors by Gloeophyllum trabeum pretreatment promotes humic substance formation during co-composting of pig manure and wheat straw. J Clean Prod. 2021;315:128211. doi: 10.1016/j.jclepro.2021.128211

[9] Chen A, Han Z, Xie X, Song C, Zhang X, Zhao Y. Co-composting sugar-containing waste with chicken manure-A new approach to carbon sequestration. J Environ Manage. 2024;356:120609. doi: 10.1016/j.jenvman.2024.120609 38498961

[10] Coşkun ÖF. Eustress and hormesis improve quality resilience and resource efficiency in horticultural crops through controlled preharvest stress management. Discov Plants. 2026;3(1). doi: 10.1007/s44372-026-00538-8

[11] Muscolo A, Papalia T, Settineri G, Mallamaci C, Jeske-Kaczanowska A. Are raw materials or composting conditions and time that most influence the maturity and/or quality of composts? Comparison of obtained composts on soil properties. Journal of Cleaner Production. 2018;195:93–101. doi: 10.1016/j.jclepro.2018.05.204

[12] Zhou Y, Selvam A, Wong JWC. Evaluation of humic substances during co-composting of food waste, sawdust and Chinese medicinal herbal residues. Bioresour Technol. 2014;168:229–34. doi: 10.1016/j.biortech.2014.05.070 24951275

[13] Lu M, Feng Q, Li X, Xu B, Shi X, Guo R. Effects of arginine modified additives on humic acid formation and microbial metabolic functions in biogas residue composting. J Environ Chem Eng. 2022;10(6):108675. doi: 10.1016/j.jece.2022.108675

[14] Wang S, Du X, Yin R, Sun H, Song B, Han Q, et al. Performance of co-composting Pholiota nameko spent mushroom substrate and pig manure at different proportions: Chemical properties and humification process. J Environ Manage. 2024;372:123325. doi: 10.1016/j.jenvman.2024.123325 39549458

[15] Sun B, Li Y, Song M, Li R, Li Z, Zhuang G, et al. Molecular characterization of the composition and transformation of dissolved organic matter during the semi-permeable membrane covered hyperthermophilic composting. J Hazard Mater. 2022;425:127496. doi: 10.1016/j.jhazmat.2021.127496 34896709

[16] Cheng A, Zhou M, Li T, Yu X, Chen D, Liu X, et al. Chemical and Molecular Profiling of Dissolved Organic Matter within Varied Compost: A Comprehensive Spectroscopic Study. Waste Biomass Valor. 2024;15(6):3749–59. doi: 10.1007/s12649-024-02423-5

[17] Qi H, Wang J, Zhang L, Chen L, Zhao Y, Wei Z. Activation effect of catechol on biotic and abiotic factors of humus formation during chicken manure composting. Waste Manag. 2022;149:146–55. doi: 10.1016/j.wasman.2022.06.014 35728478

[18] Aravind MK, Kappen J, Narayanamoorthi E, Sanjaykumar A, Varalakshmi P, Arockiadoss T, et al. Bioengineered magnetic graphene oxide microcomposites for bioremediation of chromium in ex situ - A novel strategy for aggrandized recovery by electromagnetic gadgetry. Environ Pollut. 2022;308:119675. doi: 10.1016/j.envpol.2022.119675 35753546

[19] Meng L, Li W, Zhang S, Zhang X, Zhao Y, Chen L. Improving sewage sludge compost process and quality by carbon sources addition. Sci Rep. 2021;11(1):1319. doi: 10.1038/s41598-020-79443-3 33446686 PMC7809052

[20] Yao X, Zhou H, Meng H, Ding J, Shen Y, Cheng H. Amino acid profile characterization during the co-composting of a livestock manure and maize straw mixture. J Clean Prod. 2021;278:123494. doi: 10.1016/j.jclepro.2020.123494

[21] Hill PW, Farrar JF, Jones DL. Decoupling of microbial glucose uptake and mineralization in soil. Soil Biology and Biochemistry. 2008;40(3):616–24. doi: 10.1016/j.soilbio.2007.09.008

[22] Hansen AM, Kraus TEC, Pellerin BA, Fleck JA, Downing BD, Bergamaschi BA. Optical properties of dissolved organic matter (DOM): effects of biological and photolytic degradation. Limnol Oceanogr. 2016;61:1015–32. doi: 10.1002/lno.10270

[23] Pan W, Zhou J, Tang S, Wu L, Ma Q, Marsden KA, et al. Utilisation and transformation of organic and inorganic nitrogen by soil microorganisms and its regulation by excessive carbon and nitrogen availability. Biol Fertil Soils. 2023;59(4):379–89. doi: 10.1007/s00374-023-01712-w

[24] Kong Y, Zhang J, Zhang X, Gao X, Yin J, Wang G. Applicability and limitation of compost maturity evaluation indicators: a review. Chem Eng J. 2024;489:151386. doi: 10.1016/j.cej.2024.151386

[25] Wu D, Gao W, Zhao Y, Wei Z, Song C, Qu F. Elaborating the microbial mechanism of humic substance formation during lignocellulosic biomass composting by inoculation with different functional microbes. Ind Crop Prod. 2024;208:117838. doi: 10.1016/j.indcrop.2023.117838

[26] Chen M, Huang Y, Wang C, Gao H. The conversion of organic nitrogen by functional bacteria determines the end-result of ammonia in compost. Bioresour Technol. 2020;299:122599. doi: 10.1016/j.biortech.2019.122599 31865156

[27] Mason-Jones K, Kuzyakov Y. “Non-metabolizable” glucose analogue shines new light on priming mechanisms: Triggering of microbial metabolism. Soil Biology and Biochemistry. 2017;107:68–76. doi: 10.1016/j.soilbio.2016.12.015

[28] Ji Z, Zhang L, Liu Y, Li X, Li Z. Evaluation of composting parameters, technologies and maturity indexes for aerobic manure composting: A meta-analysis. Sci Total Environ. 2023;886:163929. doi: 10.1016/j.scitotenv.2023.163929 37156376

[29] Zhang Y, Chen M, Guo J, Liu N, Yi W, Yuan Z, et al. Study on dynamic changes of microbial community and lignocellulose transformation mechanism during green waste composting. Eng Life Sci. 2022;22(5):376–90. doi: 10.1002/elsc.202100102 35573133 PMC9077819

[30] Park JW, Dec J, Kim JE, Bollag JM. Effect of humic constituents on the transformation of chlorinated phenols and anilines in the presence of oxidoreductive enzymes or birnessite. Environ Sci Technol. 1999;33:2028–34. doi: 10.1021/es9810787

[31] Matlock MM, Howerton BS, Henke KR, Atwood DA. A pyridine-thiol ligand with multiple bonding sites for heavy metal precipitation. J Hazard Mater. 2001;82(1):55–63. doi: 10.1016/s0304-3894(00)00353-8 11165061

[32] Mu D, Wang C, Geng X, Zhao Y, Mohamed TA, Wu D, et al. Effect of Maillard reaction based on catechol polymerization on the conversion of food waste to humus. Chemosphere. 2024;353:141560. doi: 10.1016/j.chemosphere.2024.141560 38417496

[33] Wang N, Zhang Q, Han W, Bai C, Hou B, Liu Y, et al. Chemical Characteristics of Dark-Brown Humic-like Substances Formed from the Abiotic Condensation of Maillard Precursors with Different Glycine Concentrations. Agronomy. 2022;12(9):2199. doi: 10.3390/agronomy12092199

[34] Liu Y, Zhang K, Zhang H, Zhou K, Chang Y, Zhan Y, et al. Humic acid and phosphorus fractions transformation regulated by carbon-based materials in composting steered its potential for phosphorus mobilization in soil. J Environ Manage. 2023;325(Pt A):116553. doi: 10.1016/j.jenvman.2022.116553 36283197

[35] Zhou Y, Zhang J, Xu L, Nadeem MY, Li W, Jiang Y, et al. Long-term fertilizer postponing promotes soil organic carbon sequestration in paddy soils by accelerating lignin degradation and increasing microbial necromass. Soil Biology and Biochemistry. 2022;175:108839. doi: 10.1016/j.soilbio.2022.108839

[36] Weber J, Jerzykiewicz M, Ukalska‐Jaruga A, Ćwieląg‐Piasecka I, Jamroz E, Kocowicz A, et al. Properties of humin isolated from Polish arable soils: The most recalcitrant fraction of soil organic matter that prevent soil degradation. Land Degrad Dev. 2024;35(7):2425–36. doi: 10.1002/ldr.5070

[37] Lin J, Mao Y, Mai L, Li G, Liu H, Peng S, et al. Accelerating the humification of mushroom waste by regulating nitrogen sources composition: Deciphering mechanism from bioavailability and molecular perspective. Chemosphere. 2024;349:140816. doi: 10.1016/j.chemosphere.2023.140816 38040259

[38] Mohammed I, Kodaolu B, Zhang T, Wang Y, Audette Y, Longstaffe J. Analysis of Molecular Structure Changes in Humic Acids from Manure-Amended Soils over 17 Years Using Elemental Analysis and Solid-State 13C Nuclear Magnetic Resonance Spectroscopy. Soil Systems. 2023;7(3):76. doi: 10.3390/soilsystems7030076

[39] Bui VKH, Truong HB, Hong S, Li X, Hur J. Biotic and abiotic catalysts for enhanced humification in composting: a comprehensive review. J Clean Prod. 2023;402:136832. doi: 10.1016/j.jclepro.2023.136832

[40] Blodgett KN, Fischer JL, Zwier TS, Sibert EL. The missing NH stretch fundamental in S1 methyl anthranilate: IR-UV double resonance experiments and local mode theory. Phys Chem Chem Phys. 2020;22(25):14077–87. doi: 10.1039/d0cp01916j 32568351

[41] Martín AP-S, Marhuenda-Egea FC, Bustamante MA, Curaqueo G. Spectroscopy Techniques for Monitoring the Composting Process: A Review. Agronomy. 2023;13(9):2245. doi: 10.3390/agronomy13092245

[42] Fersi M, Hajji R, Mbarki K, Louati I, Jedidi N, Hassen A, et al. Spectroscopic and microscopic characterization of humic acids from composts made by co-composting of green waste, spent coffee and OMWW sludge. Environ Technol. 2024;45(27):6024–37. doi: 10.1080/09593330.2024.2320622 38403904

[43] Amir S, Jouraiphy A, Meddich A, El Gharous M, Winterton P, Hafidi M. Structural study of humic acids during composting of activated sludge-green waste: elemental analysis, FTIR and 13C NMR. J Hazard Mater. 2010;177(1–3):524–9. doi: 10.1016/j.jhazmat.2009.12.064 20106591

[44] Li S, Li D, Li J, Li G, Zhang B. Evaluation of humic substances during co-composting of sewage sludge and corn stalk under different aeration rates. Bioresour Technol. 2017;245(Pt A):1299–302. doi: 10.1016/j.biortech.2017.08.177 28899677

[45] Ma Y, Shang X, Zhao Y, Lu X, Liu X. Insights into organic matter evolution during food waste stabilization induced by 14-hour high-temperature fermentation. Environ Sci Pollut Res Int. 2023;30(12):35064–75. doi: 10.1007/s11356-022-24717-8 36522576

