# Supplementary material for: Differential regulation of humification by Maillard reaction precursors during the co-composting of pig manure and spent mushroom substrate
Source: PLoS One. 2026 Nov 11;21(11):e0361892. doi: 10.1371/journal.pone.0361892 (PMC13754110; doi:10.1371/journal.pone.0361892)
Supplement: Supplementary file 1 [file pone.0361892.s001.docx]

**S1 Table. Fluorescence indices of DOM obtained at the initial and final stages of simulated mesophilic PM-SMS co-composting incubation**

| **Treatments** | **Composting duration (d)** | **FI** | **BIX** | **HIX** |
| --- | --- | --- | --- | --- |
|  |  |  |  |  |
| CK | 0 | 1.53 ± 0.12c | 0.171 ± 0.07c | 11.5 ± 1.43de |
|  | 120 | 1.91 ± 0.14ab | 0.348 ± 0.05b | 19.8 ± 1.68b |
| Cat | 0 | 1.72 ± 0.16b | 0.239 ± 0.09bc | 9.2 ± 0.92g |
|  | 120 | 2.03 ± 0.15a | 0.469 ± 0.05a | 5.8 ± 0.41h |
| Glu | 0 | 1.46 ± 0.11c | 0.143 ± 0.09d | 12.1 ± 1.51d |
|  | 120 | 1.79 ± 0.08b | 0.278 ± 0.07bc | 25.7 ± 2.08a |
| Gly | 0 | 1.51 ± 0.13c | 0.167 ± 0.08c | 9.9 ± 0.92ef |
|  | 120 | 1.42 ± 0.09c | 0.165 ± 0.07cd | 14.5 ± 1.65c |
