# Supplementary material for: Differential regulation of humification by Maillard reaction precursors during the co-composting of pig manure and spent mushroom substrate
Source: PLoS One. 2026 Nov 11;21(11):e0361892. doi: 10.1371/journal.pone.0361892 (PMC13754110; doi:10.1371/journal.pone.0361892)
Supplement: Supplementary file 2 [file pone.0361892.s002.docx]

**S2 Table. FTIR relative intensities (% of total area) for the HA as influenced by different MRPs**

| **Treatments** | **Composting duration (d)** | **FTIR relative intensities (cm^-1^)** | | | | | | |
| --- | --- | --- | --- | --- | --- | --- | --- | --- |
|  |  | **3401-3433** | **2921-2968 ^a^** | **2847-2873 ^b^** | **1564-1596 ^d^** | **1407-1461 ^c^** | **1033-1052** | **[(a+b+c)/d]** |
| CK | 0 | 43.3 | 9.08 | 4.83 | 15.2 | 8.1 | 19.4 | 1.45 |
|  | 120 | 42.5 | 8.43 | 4.11 | 17.9 | 7.2 | 18.9 | 1.10 |
| Cat | 0 | 46.4 | 10.3 | 3.71 | 15.5 | 3.68 | 20.1 | 1.14 |
|  | 120 | 45.9 | 8.95 | 2.85 | 19.8 | 2.98 | 19.2 | 0.75 |
| Glu | 0 | 48.1 | 8.80 | 4.03 | 13.7 | 5.57 | 19.6 | 1.34 |
|  | 120 | 45.7 | 7.08 | 3.32 | 18.7 | 5.22 | 19.1 | 0.84 |
| Gly | 0 | 46.5 | 9.70 | 3.61 | 14.8 | 5.13 | 19.4 | 1.25 |
|  | 120 | 44.2 | 8.38 | 3.04 | 21 | 4.31 | 18.2 | 0.75 |
