# Supplementary material for: Differential regulation of humification by Maillard reaction precursors during the co-composting of pig manure and spent mushroom substrate
Source: PLoS One. 2026 Nov 11;21(11):e0361892. doi: 10.1371/journal.pone.0361892 (PMC13754110; doi:10.1371/journal.pone.0361892)
Supplement: Supplementary file 3 [file pone.0361892.s003.docx]

**S3 Table. Eigenvalues of the three PCs, along with their individual and cumulative contribution rates**

| **Principal component** | **Eigenvalue** | **Contribution rate/%** | **Cumulative contribution rate/%** |
| --- | --- | --- | --- |
| 1 | 7.211 | 55.472 | 55.472 |
| 2 | 3.629 | 27.915 | 83.387 |
| 3 | 2.16 | 16.613 | 100.000 |
