# Supplementary material for: Differential regulation of humification by Maillard reaction precursors during the co-composting of pig manure and spent mushroom substrate
Source: PLoS One. 2026 Nov 11;21(11):e0361892. doi: 10.1371/journal.pone.0361892 (PMC13754110; doi:10.1371/journal.pone.0361892)
Supplement: Supplementary file 4 [file pone.0361892.s004.docx]

**S4 Table. Load matrix and eigenvector matrix of each PC**

| **Index** | **Loading coefficient** | | | **Linear combination coefficient matrix** | | |
| --- | --- | --- | --- | --- | --- | --- |
|  | **PC1** | **PC2** | **PC3** | **PC1** | **PC2** | **PC3** |
| Total nutrient | 0.59 | 0.656 | 0.47 | 0.082 | 0.181 | 0.218 |
| OM | 0.59 | 0.656 | 0.47 | 0.082 | 0.181 | 0.218 |
| C_WSS_ | -0.61 | -0.679 | 0.409 | -0.085 | -0.187 | 0.189 |
| C_HE_ | 0.843 | 0.532 | 0.079 | 0.117 | 0.147 | 0.036 |
| C_HA_ | -0.927 | 0.006 | 0.376 | -0.128 | 0.002 | 0.174 |
| C_Hu_ | 0.99 | 0.025 | -0.139 | 0.137 | 0.007 | -0.064 |
| EC | -0.347 | 0.371 | -0.861 | -0.048 | 0.102 | -0.399 |
| GI | 0.842 | -0.463 | 0.276 | 0.117 | -0.128 | 0.128 |
| DP | -0.96 | 0.046 | 0.276 | -0.133 | 0.013 | 0.128 |
| C/N ratio | -0.909 | 0.311 | -0.277 | -0.126 | 0.086 | -0.128 |
| O/C ratio | 0.891 | -0.33 | -0.313 | 0.124 | -0.091 | -0.145 |
| H/C ratio | -0.429 | 0.786 | 0.446 | -0.059 | 0.217 | 0.206 |
| (a+b+c)/d | -0.195 | 0.92 | -0.34 | -0.027 | 0.254 | -0.158 |
