# Supplementary material for: Differential regulation of humification by Maillard reaction precursors during the co-composting of pig manure and spent mushroom substrate
Source: PLoS One. 2026 Nov 11;21(11):e0361892. doi: 10.1371/journal.pone.0361892 (PMC13754110; doi:10.1371/journal.pone.0361892)
Supplement: Supplementary file 5 [file pone.0361892.s005.docx]

**S5 Table. Average score and total score of main components of adding different MRPs**

| **Composting duration (d)** | **Treatments** | **F_1_** | | **F_2_** | | **F_3_** | | **Comprehensive evaluation** | |
| --- | --- | --- | --- | --- | --- | --- | --- | --- | --- |
|  |  |  |  |  |  |  |  |  |  |
|  |  | **Score** | **Sort** | **Score** | **Sort** | **Score** | **Sort** | **Score** | **Sort** |
|  |  |  |  |  |  |  |  |  |  |
| 120 d | CK | -1.416 | 4 | 0.343 | 2 | -0.358 | 3 | -0.749 | 4 |
|  | Cat | 0.079 | 3 | -0.338 | 3 | 1.459 | 1 | 0.192 | 2 |
|  | Glu | 0.888 | 1 | 1.174 | 1 | -0.29 | 2 | 0.772 | 1 |
|  | Gly | 0.449 | 2 | -1.179 | 4 | -0.811 | 4 | -0.215 | 3 |
